# Design, Facilitation, and Evaluation of Tabletop Exercises for Prehospital Mass Casualty Preparedness: Scoping Review

**DOI:** 10.2196/96228

**Published:** 2026-10-05

**Authors:** Paurnami Prashanth, Ali AlRahma, Dayol Narayanan, Abu Omayer, Azza Yousif, Ives Hubloue, Nabil Zary

**Affiliations:** 1 Emergency Department, Dubai Health, Umm Hurair Second, Bur Dubai, Dubai, United Arab Emirates, 971 800 60 ext 800; 2 Dubai Corporation for Ambulance Services, Dubai, United Arab Emirates; 3 Institute of Learning, Mohammed Bin Rashid University of Medicine and Health Sciences, Dubai Health, Dubai, United Arab Emirates; 4 Emergency Department, UZ Brussel University Hospital, Brussels, Belgium; 5 Research Group on Emergency and Disaster Medicine (ReGEDiM) Vrije Universiteit Brussel, Brussels, Belgium

**Keywords:** tabletop exercise, mass casualty incident, prehospital, scoping review, Kirkpatrick Evaluation Model, simulation-based education, disaster preparedness, applied learning, training transfer, exercise design

## Abstract

**Background:**

Tabletop exercises (TTXs) are commonly used to enhance prehospital readiness for mass casualty incidents (MCIs). However, evidence on their design, facilitation, and evaluation remains scattered. TTXs simulate organized interactions at 3 levels: among individual responders and response protocols, within interdisciplinary teams, and across organizations and systems. While existing reviews cover tabletop simulation generally, they do not specifically focus on the prehospital MCI interface or assess whether evaluation methods match the exercise objectives.

**Objective:**

This scoping review aimed to explore how TTXs are designed, facilitated, and evaluated in prehospital MCI preparedness. It classified outcomes using the Kirkpatrick Evaluation Model and analyzed how evaluation methods align with the purpose of each exercise.

**Methods:**

We performed a scoping review following Arksey and O’Malley’s framework, with enhancements from Levac et al, along with PRISMA-ScR (Preferred Reporting Items for Systematic Reviews and Meta-Analyses Extension for Scoping Reviews) and PRISMA-S (PRISMA Extension for Reporting Literature Searches) guidelines. Searches across PubMed, Embase, Scopus, PsycINFO, CINAHL, the Cochrane Library, ClinicalTrials.gov, Google Scholar, and references were limited to English peer-reviewed and gray literature published from 2015 to May 2026. Eligible studies focused on TTXs related to prehospital disaster or MCI preparedness and reported measurable educational, clinical, or system outcomes. The review protocol was registered beforehand on Protocols.io. Two reviewers (PP and AA) independently screened records, extracted data, and classified outcomes using Kirkpatrick levels, with an added level 2+ (Applied Learning) category for structured performance evaluation during exercises.

**Results:**

Thirteen studies from 9 countries were included. They were categorized into a preliminary 3-tier typology called the TTX Design Spectrum, representing increasing levels of interaction: algorithm-rehearsal exercises (n=2) focused on individual responders’ interaction with triage protocols, scenario-based decision-training exercises (n=7) aimed at multidisciplinary team decision-making under realistic conditions, and systems integration exercises (n=4) centered on interagency coordination and system-level preparedness. Facilitation varied by exercise purpose, from standardized assessment-focused approaches to expert-led and multidisciplinary facilitation. Evaluation primarily targeted Kirkpatrick level 1: Reaction (n=10), level 2: Learning (n=10), and level 2+ (Applied Learning) (n=8), with fewer studies examining level 3: Behavior (n=2) or level 4: Results (n=3). Operational frameworks were reported more consistently than formal educational design or assessment frameworks. Additionally, natural disaster scenarios and evidence from resource-limited settings were underrepresented.

**Conclusions:**

TTXs for prehospital MCI preparedness should be viewed as a collection of related exercise types rather than a single, uniform intervention. This review introduces an initial typology called the TTX Design Spectrum, along with the level 2+ (Applied Learning) classification, which operationalizes the distinction between in-training performance and behavioral transfer. These tools aim to help align exercise purpose, facilitation, and evaluation strategies. Future research should focus on validating this typology, enhancing follow-up assessments of behavioral transfer, and adapting TTX design for natural disaster and resource-limited settings.

## Introduction

Mass casualty incidents (MCIs) and disasters continue to challenge health systems because they demand quick coordination, prioritization, and adaptation amid uncertainty, time constraints, and limited resources. The Sendai Framework for Disaster Risk Reduction emphasizes the importance of preparedness for effective response in modern disaster management [1]. Prehospital readiness involves personnel and systems managing triage, incident command, scene coordination, interagency communication, resource management, and treatment prioritization. This preparedness significantly impacts outcomes when systems are overwhelmed. As a result, health care simulation use has increased [2,3], and recent tabletop and disaster education research highlights incident command, triage, surge management, communication, and resource allocation as essential skills that require practice before real emergencies [4-6].

Tabletop exercises (TTXs) stand out among simulation methods because they are structured, discussion-driven activities where participants can rehearse plans, roles, communication, and operational choices without the logistical challenges of full-scale or functional exercises [5,7]. They are also versatile, being customizable to an organization’s specific risks with adaptable goals, scope, and difficulty levels [2,5], making them a popular tool for improving disaster and MCI preparedness [4,8-10].

The existing literature indicates that TTXs are not a uniform type of intervention. Some primarily concentrate on practicing triage algorithms, while others focus on team decision-making, interagency communication, or organizational coordination [2,4-6]. This variation is expected because exercises serve different educational and operational goals. The challenge lies in the fact that the term “tabletop exercise” is often used to describe quite different activities. Without a common framework for describing the purpose of each exercise, educators and planners struggle to determine whether an evaluation method fits the exercise’s objectives and find it difficult to compare findings across studies that use the same term for different types of activities.

At their core, TTXs are organized interactions involving individual responders, the protocols they follow, professionals from various disciplines, and different organizations, agencies, and systems. The specific level of interaction an exercise aims to rehearse determines who participates, how it is facilitated, and what metrics should be used for evaluation. Consequently, a typology of exercise purposes also reflects the types of interactions being trained. This shared vocabulary helps determine whether an evaluation method is appropriate for a particular exercise and allows for comparison of evidence across studies.

Recent review literature highlights the need for a more targeted synthesis. Frégeau et al [7] conducted a comprehensive scoping review of tabletop simulations in medical emergencies, analyzing 70 studies across various settings, specialties, formats, and learner groups. They found that reported outcomes mainly focused on the Kirkpatrick reaction and learning levels [7]. Emaliyawati et al [8] reviewed 12 tabletop disaster exercise studies involving health care workers and students, concluding that TTXs enhance knowledge, attitudes, preparedness, confidence, and performance. Although these reviews are valuable, they address TTXs broadly, rather than focusing specifically on the prehospital interface. They do not analyze how prehospital MCI competencies are distributed across different exercise designs, nor do they systematically link exercise purpose with facilitation style and evaluation depth. As far as we know, no published review has specifically synthesized TTXs for prehospital MCI preparedness while examining the alignment among exercise design, facilitation, and evaluation [7,8].

This scoping review aimed to map the existing literature and clarify how TTXs function within prehospital MCI preparedness, considering them as a group of related but distinct interventions. The objectives included systematically investigating how TTXs have been used to evaluate or enhance prehospital readiness for MCIs; classifying reported outcomes according to the Kirkpatrick Evaluation Model, including structured performance within exercises; describing key design, delivery, and facilitation features along with the competencies they target; and pinpointing gaps in the evidence. The review seeks to develop a preliminary typology called the TTX Design Spectrum, which aims to guide purpose-aligned design, facilitation, and evaluation in prehospital disaster preparedness.

## Methods

### Study Design and Methodological Framework

#### Overview

This scoping review, part of the Mass-Casualty Incident, Prehospital Emergency Response project, systematically maps evidence related to TTXs for prehospital disaster preparedness. Our methodology follows Arksey and O’Malley’s [11] framework, with modifications by Levac et al [12], and adheres to PRISMA-ScR (Preferred Reporting Items for Systematic Reviews and Meta-Analyses Extension for Scoping Reviews) reporting standards [13]. The protocol was registered on Protocols.io (July 3, 2025) [14] and published as a preprint [15], ensuring transparency and reproducibility. Stakeholder consultation, recommended by Levac et al [12] for knowledge translation, was not carried out because the study primarily focused on evidence mapping for research synthesis rather than developing practice guidelines.

#### Stage 1: Identifying the Research Question

This scoping review was based on 2 main research questions and 3 subsidiary questions. The primary questions explored how TTXs are used to assess or enhance prehospital preparedness for MCIs, and which Kirkpatrick evaluation levels (Reaction, Learning, Behavior, and Results) are most frequently reported in prehospital MCI TTX studies. The secondary questions investigated the key design and delivery features of these TTXs, the competencies and operational areas they target, and identified gaps in evidence concerning evaluation methods and their effectiveness.

#### Stage 2: Identifying Relevant Studies

Search reporting followed the PRISMA-S (PRISMA Extension for Reporting Literature Searches) extension for literature search reporting [16], along with PRISMA-ScR. A completed PRISMA-ScR checklist is included in Checklist 1. We searched several multidisciplinary databases, including PubMed, Embase, Scopus, PsycINFO (via APA PsycNet), CINAHL, Cochrane Library, and ClinicalTrials.gov, to cover biomedical, educational, and allied health research. Each database and registry was searched individually; no multidatabase search was performed simultaneously. We also reviewed Google Scholar for gray literature and manually screened reference lists of included studies and relevant reviews. No contact was made with study authors, experts, manufacturers, or external contacts to gather additional records or data. Our search methods included only database searches, Google Scholar, and manual reference list screening.

On June 10, 2025, we established a detailed search strategy based on the Population-Concept-Context framework (Table 1), which was subsequently updated on May 15, 2026. The Population targeted prehospital health care workers such as paramedics, emergency medical technicians, emergency physicians, and nurses, as well as emergency system administrators involved in MCI responses. The Concept focused on TTXs used for training and preparedness, with outcomes measurable at the educational, clinical, or system levels. The Context covered prehospital systems that respond to mass-casualty incidents and disasters across various geographic areas.

**Table 1.** Search strategy overview using the Population-Concept-Context framework. Representative controlled vocabulary and free-text terms are displayed. Detailed database-specific search strings are available in Multimedia Appendix 1.

Category	Keywords	Search strategy
Population	“Emergency Medical Services” [MeSH]; “Emergency Medical Technicians” [MeSH]; “Allied Health Personnel” [MeSH]	paramedic*; “first responder*”; “first responder”; “first responders”; ambulance*; “emergency medical service*”; “emergency medical service”; “emergency medical services”; EMS^a^
Concept	“Simulation Training” [MeSH]; “Patient Simulation” [MeSH]; “Computer Simulation” [MeSH]; “simulation training”; “tabletop simulation”; “tabletop exercise”	tabletop exercise*; table-top exercise*; tabletop simulation*; tabletop drill*; discussion-based exercise*; scenario-based simulation*; preparedness exercise*; board game*; simulation game*; gamified exercise; paper-based exercise
Context	“Mass Casualty Incidents” [MeSH]; “Disaster Planning” [MeSH]; “Emergency Preparedness” [MeSH]; “Triage” [MeSH]; “mass disaster”; “field triage”; “incident command”; “prehospital care”	emergency preparedness; disaster preparedness; mass casualty incident*; MCI^b^; field triage; incident command; prehospital care; disaster simulation; response coordination; crisis; disaster response; multi-casualty; multiple casualty

a EMS: emergency medical services.

b MCI: mass casualty incident.

We used a combination of controlled vocabulary (MeSH) and free-text terms with Boolean operators in search strings tailored to each database, refining them iteratively to improve sensitivity and specificity (Table 1). An information specialist or librarian peer-reviewed the search strategy following the Peer Review of Electronic Search Strategies criteria before final implementation [17]. No published search filters were used, nor were previous review strategies reused or adapted; instead, prior reviews helped identify relevant references through citation screening. Details about platforms and complete database-specific search strings, including the full PubMed strategy, are available in Multimedia Appendix 1.

#### Stage 3: Study Selection

Retrieved records from various sources, including citation searches and gray literature, were imported into Covidence [18], where they were pooled and deduplicated before screening. Two reviewers, PP and AA, independently examined all records through a 2-stage process: first screening titles and abstracts and then reviewing full texts. Any disagreements were resolved through discussion or, if necessary, by consulting a third reviewer. These disagreements were systematically recorded in Covidence and addressed through structured discussions with documented reasons.

### Eligibility Criteria

#### Overview

We included studies that assessed TTXs in prehospital disaster or MCI preparedness and reported measurable educational, clinical, or system outcomes. These were peer-reviewed papers or gray literature published in English between January 2015 and May 2026. For this review, “prehospital” was mainly defined by the medical phase and operational functions of MCI or disaster response, rather than solely by participant role or physical setting. This encompassed EMS (emergency medical services) care, field triage, scene coordination, casualty distribution, transport decisions, and the transition from prehospital to hospital care. Eligible study designs included quantitative, qualitative, mixed methods, quasi-experimental, pre-post, randomized, observational, descriptive, pilot, feasibility, and implementation studies. We excluded systematic reviews, narrative reviews, editorials, commentaries, opinion pieces, conference abstracts without full texts, studies lacking full-text access, non-English studies, studies published before 2015, and studies where TTXs were not the primary focus. Additionally, studies focused on nonmedical TTX applications, such as military, corporate, cybersecurity, or other non–health care settings, were excluded unless they included a relevant medical prehospital or MCI response component. Studies involving hospital personnel were included only if they addressed prehospital medical functions, EMS coordination, transport, casualty distribution, or the hospital transition process.

We chose papers from 2015 onward because initial scoping indicated limited peer-reviewed literature before 2015 on TTX outcomes in prehospital MCI settings. Furthermore, reporting standards for simulation-based education improved significantly during this period [19], and the 2015 Sendai Framework for Disaster Risk Reduction [1] provided a current policy context for disaster preparedness research.

#### Stage 4: Data Charting

Before starting the formal data extraction, we tested a standardized data-charting form with 2 reviewers (DN and AO) across 5 studies to verify that the extraction fields were clear, comprehensive, and consistently understood. During this pilot, reviewers independently extracted data, compared their entries, discussed any discrepancies, and refined the form before proceeding with full extraction. The finalized form included study details (ID, country, design, population, and sample size), TTX features (targeted competencies, duration, scenario design, and preparatory instruction), facilitation aspects, evaluation methods, Kirkpatrick levels addressed, and the underlying training, assessment, and operational frameworks.

After the data-charting form was finalized, 2 reviewers (DN and AO) independently extracted data from all included studies. They compared their extractions, resolving any discrepancies through discussion and consensus. If they could not reach an agreement, a third reviewer was consulted. Microsoft Excel was used to manage, review, and organize the extracted data.

Outcomes were categorized using the Kirkpatrick Evaluation Model [20,21]. Beyond the 4 standard levels, we added a level 2+ (Applied Learning) subcategory to differentiate structured performance assessments within the exercise setting from knowledge acquisition and actual behavioral transfer in real-world contexts. This subcategory helps operationalize, for TTX outcome mapping, distinctions outlined in earlier frameworks: the updated (“New World”) Kirkpatrick model’s focus on skills shown during training [22], Miller’s “shows how” level of assessment [23], and translational science models of simulation-based education outcomes (T1-T3), which differentiate in-simulation performance from later behavior and patient or system results [24]. Each outcome was classified independently; thus, a single study could contribute to multiple Kirkpatrick levels. Detailed definitions, decision rules, and examples for classification are available in Multimedia Appendix 2.

#### Stage 5: Collating, Summarizing, and Reporting the Results

We used a combination of descriptive and narrative approaches appropriate for scoping reviews. Quantitative information was summarized using frequencies and counts, while qualitative and contextual data, such as exercise design, scenario features, facilitation processes, targeted skills, and evaluation techniques, were synthesized narratively. The extracted data were categorized into 7 domains: general study details, population traits, TTX features and elements, facilitator responsibilities, data collection methods, training results, and frameworks that inform training design, assessment, and operational practice.

Through iterative cross-study comparison, we discovered common patterns in exercise design, facilitation, and evaluation. To categorize these patterns, we created the TTX Design Spectrum, which classifies exercises into 3 tiers based on their primary purpose and scope: tier 1 focuses on algorithm rehearsal, tier 2 on scenario-based decision training, and tier 3 on systems integration testing. Facilitation methods and evaluation patterns were summarized descriptively rather than formalized into additional models. This typology was developed inductively during synthesis rather than being predefined; it is an initial organizational tool and not a validated framework. Definitions can be found in Multimedia Appendix 2.

### Protocol Deviations

This review was based on a prospectively registered protocol [14,15]. Two post hoc analytical refinements were introduced: the creation of the TTX Design Spectrum typology, which provides a descriptive overview of facilitation and evaluation patterns during stage 5, and the addition of the level 2+ (Applied Learning) subcategory in outcome classification during stage 4. Neither of these changes was specified in the protocol; both were developed through iterative comparison and synthesis of data. Importantly, there were no deviations that affected the search strategy or eligibility criteria.

### Ethical Considerations

This scoping review summarized publicly accessible, previously published literature without direct involvement of human participants. Therefore, ethical approval was not required. We adhered to established ethical research principles, such as transparent reporting, proper attribution and citation, and preventing plagiarism and duplicate publications.

## Results

### Literature Search and Study Selection

Our search identified 2693 records from databases and trial registers, along with 20 additional records from other sources. After removing 272 duplicates, we screened 2441 records by title and abstract, excluding 2353. We attempted to retrieve 88 full-text reports; of these, 19 were conference abstracts without full publications. Metadata for these 19 records (author, year, title, and source) is provided in Multimedia Appendix 3. The publication years and geographic origins of unretrieved records were similar to those of the retrieved records (Multimedia Appendix 3). Of the 69 full-text reports assessed for eligibility, 56 were excluded (wrong setting, n=22; wrong intervention, n=21; wrong study design, n=11; and non-English, n=2). Thirteen studies met the inclusion criteria and were included in the final analysis. Figure 1 illustrates the study selection process.

**Figure 1.**
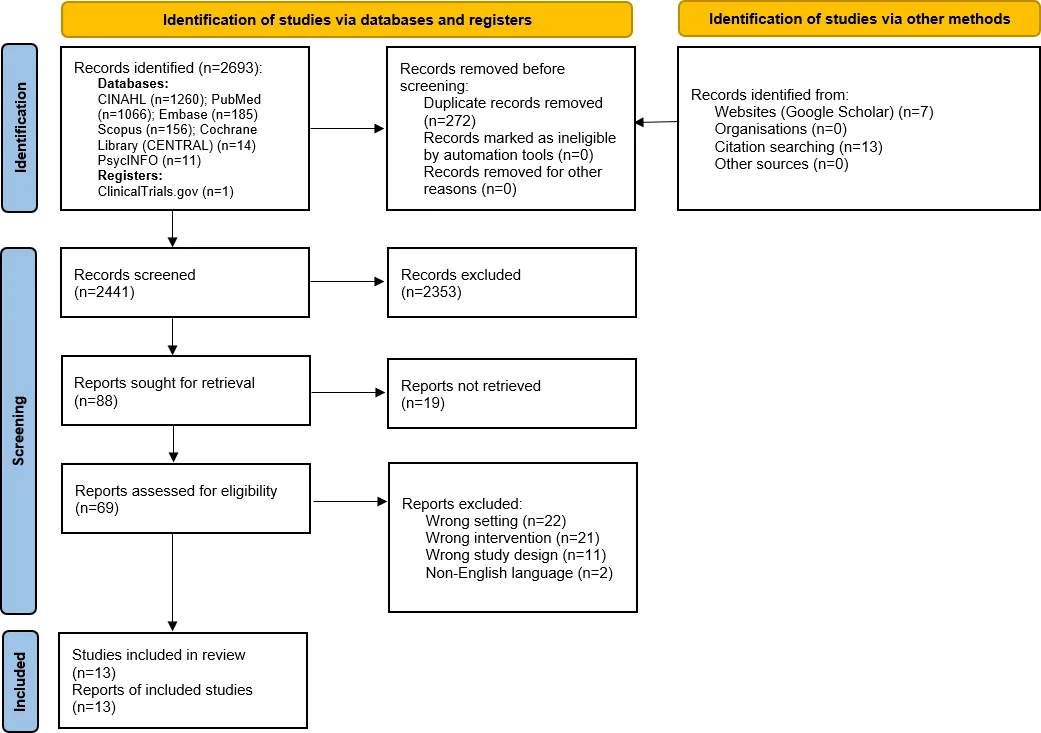
PRISMA (Preferred Reporting Items for Systematic Reviews and Meta-Analyses) 2020 flow diagram for study selection. Records were identified through 6 databases, 1 register, and other methods. Of these, 13 studies met all inclusion criteria and were categorized into 3 tiers of the Tabletop Exercise Design Spectrum.

### Study Characteristics

Thirteen studies published between 2016 and 2026 were included, with most publications in 2020 (n=3). The studies took place in various countries: 4 in the United States, 2 in Thailand, and 1 each in the United Kingdom, Saudi Arabia, South Korea, Qatar, Spain, Sweden, and Japan. The research designs comprised 4 pre-post evaluations, 3 quasi-experimental studies, 2 reliability assessments, 2 mixed methods investigations, 1 randomized controlled trial, and 1 descriptive report. The sample sizes varied from 12 to 230 participants; 1 study did not specify the number of fellows involved (refer to Table 2, footnote).

**Table 2.** Characteristics of Included Studies (N=13).

Study	Country	Design	Participants, n	Population	Duration	Scenarios
Tier 1: algorithm rehearsal (n=2)						
Cheng et al (2022) [25]	United States	Reliability study	107	Prehospital EMS^a^ professionals	4‐11 minutes per set	1 (pediatric bus crash, 25 patients; 6 triage algorithms compared)
McGlynn et al (2020) [26]	United States	Reliability study	NR^b^	Pediatric emergency medicine fellows	Not reported	3 (10-, 100-, and 1000-victim MCI^c^ scenarios; 253 patient cases)
Tier 2: scenario-based decision training (n=7)						
Kim et al (2021) [27]	South Korea	Pre-post	40	Physicians, nurses, and public health officers	25 minutes + 10 minutes debrief	1 (hydrofluoric acid explosion at chemical plant)
Phattharapornjaroen et al (2020) [28]	Thailand	Pre-post	52	Emergency physicians	2-day course	3 (building fire, terrorist bombing, and riots or active shooter)
Sena et al (2021) [4]	United States	Pre-post	18	Emergency medicine residents	2 hours	1 (explosion or mass shooting with fire)
Farhat et al (2022) [29]	Qatar	Pre-post	12	Physicians, nurses, and paramedics	3 hours	4 (biological, chemical, radiological, and nuclear CBRNE^d^)
Alakrawi et al (2024) [30]	Saudi Arabia	Quasi-experimental	45	Senior paramedic students	Not reported	1 (multivehicle accident, 15 patients)
Chumvanichaya et al (2025) [31]	Thailand	RCT^e^	83	Paramedic students	40 minutes	3 (10 simulated victims: SIEVE^f^, SORT^g^, and START^h^ protocols)
Cuartas-Alvarez et al (2026) [32]	Spain	Quasi-experimental	27	Primary care professionals (doctors and nurses)	2 hours	1 (MCI scenario: collapse with multiple victims; PHCT^i^-led prehospital response with ECC^j^ or resource coordination)
Tier 3: systems integration testing (n=4)						
Anan et al (2016) [33]	Japan	Quasi-experimental	230	Physicians, firefighters, police officers, and emergency responders	60‐85 minutes per scenario	3 (fire → chemical recognition → decontamination → triage)
Cicero et al (2019) [34]	United States	Descriptive	27	EMS, nurses, physicians, hospital administrators, and emergency managers	Not reported	1 (school bus rollover, pediatric MCI)
Skryabina et al (2020) [35]	United Kingdom	Mixed methods	125	Prehospital, hospital staff, and emergency planners	Not reported	1 (suicide bombing + marauding terrorist firearm attack)
Zimmerman et al (2026) [36]	Sweden	Mixed methods	16	Physicians, residents, specialists across emergency medicine, anesthesiology, internal medicine, surgery, psychiatry, radiology, and ophthalmology	Not reported	Not reported

a EMS: emergency medical services.

b The participant number is not clearly reported (NR); there were 253 patient cases available for triage across the scenario sets.

c MCI: mass casualty incident.

d CBRNE: Chemical, Biological, Radiological, Nuclear, Explosive.

e RCT: randomized controlled trial.

f SIEVE: Sieve (UK Major Incident Primary Triage).

g SORT: Secondary Triage (UK Major Incident).

h START: Simple Triage and Rapid Treatment.

i PHCT: primary health care team.

j ECC: emergency coordination center.

The study populations were diverse, comprising EMS personnel, paramedics, paramedic students, physicians, emergency medicine residents, pediatric emergency specialists, nurses, and public health officers. Additionally, 3 studies specifically included nonclinical stakeholders: hospital administrators and emergency managers [34], firefighters and police officers [33], and emergency planners [35]. Table 2 provides a summary of the main study characteristics, while Figure 2A and B illustrate the distribution of the studies over time and across different regions.

**Figure 2.**
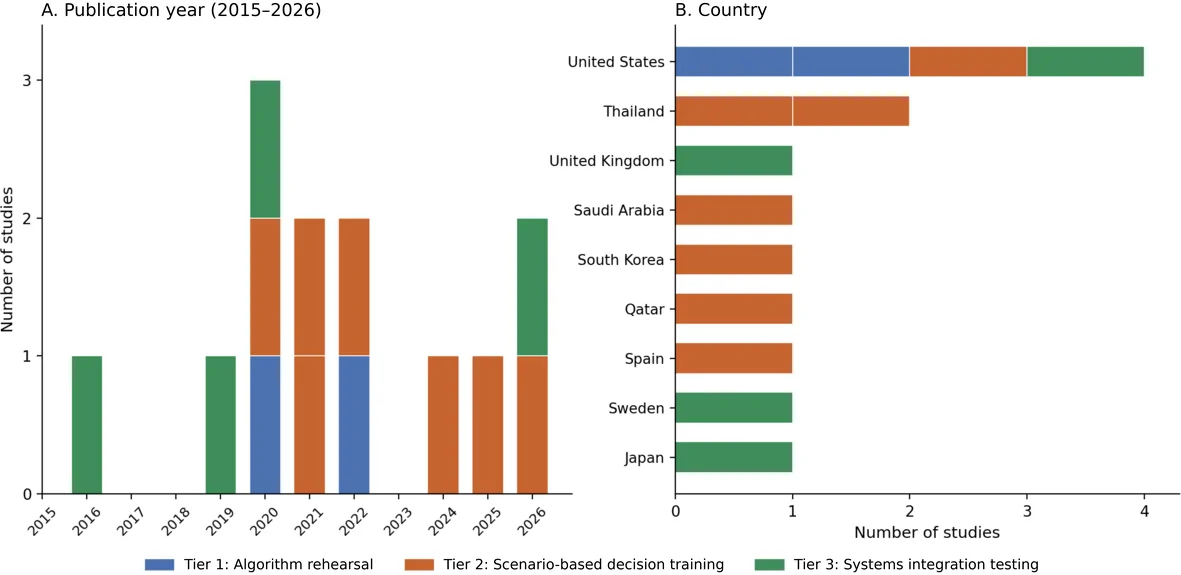
Temporal and geographic distribution of included studies (N=13). Panel A shows the count of included studies by publication year from 2015 to 2026, while panel B illustrates the geographic distribution across countries. In both panels, the bar segments are color-coded to represent the Tabletop Exercise Design Spectrum tiers: tier 1 for algorithm rehearsal, tier 2 for scenario-based decision training, and tier 3 for systems integration testing.

### Organization of the Synthesis

Figure 3 offers a visual overview that clarifies the relationship between the classification systems and how the results are organized. In this review, “tiers” specifically refer to the TTX Design Spectrum, which relates to exercise purpose and scope, while “levels” exclusively denote Kirkpatrick outcome levels, indicating evaluation depth. Facilitation modes describe how facilitators engaged participants. In the discussion, we use descriptive names such as algorithm rehearsal, scenario-based decision training, and systems integration testing to refer to tiers, thereby keeping the 2 numbering systems distinct.

**Figure 3.**
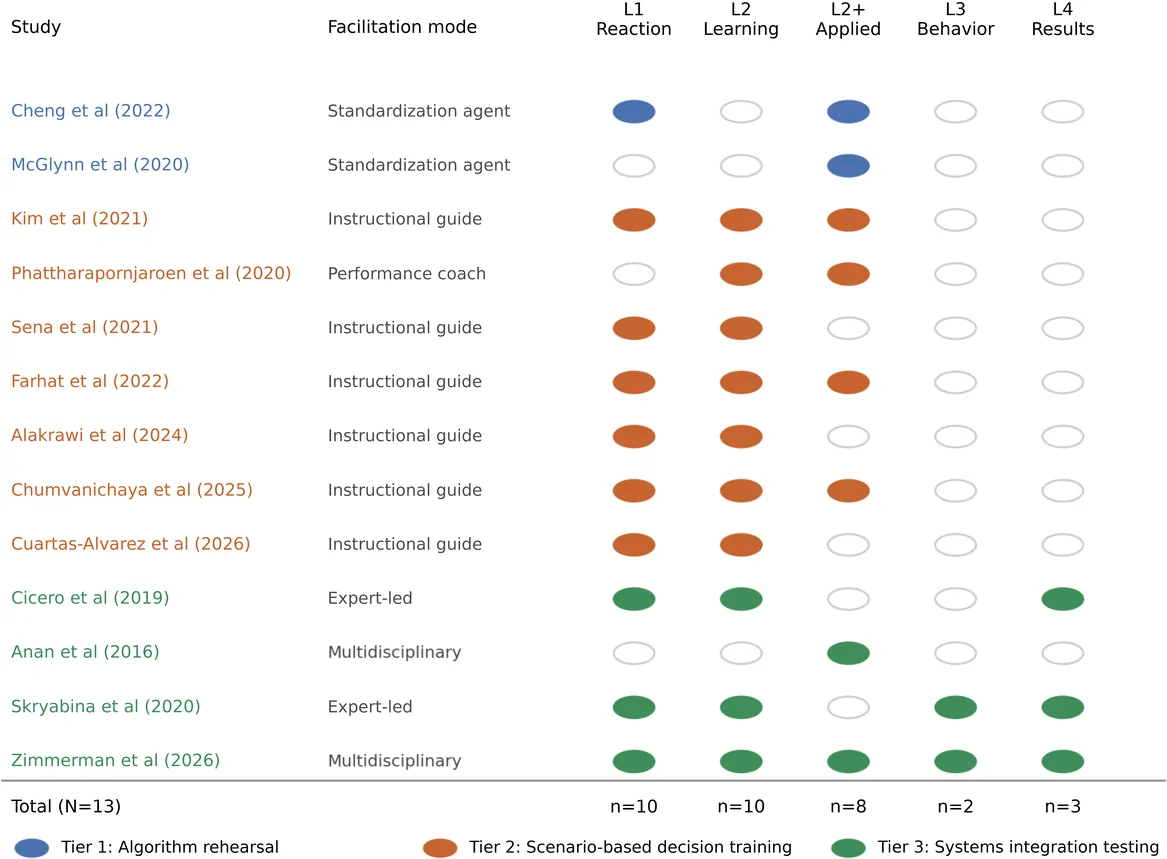
Study-level matrix of tabletop exercise (TTX) tier, primary facilitation mode, and Kirkpatrick levels assessed (N=13). Each row represents a study included, color-coded by TTX Design Spectrum tier, highlighting its main facilitation mode and the Kirkpatrick outcome levels evaluated (level 1, Reaction; level 2, Learning; level 2+, Applied Learning; level 3, Behavior; and level 4, Results). Filled circles denote assessed levels; column totals match those in Table 3. Each study is assigned to 1 tier and 1 primary facilitation mode but can contribute to multiple outcome levels [4,25-36].

### The TTX Design Spectrum: 3 Tiers of Exercise Purpose

#### Overview

Through iterative comparison across studies, we grouped the 13 included studies into 3 initial tiers based on each TTX’s primary goal and scope. This grouping was consistent across 3 key dimensions: learning objectives, participant makeup, and outcome focus. Tier 1 involved proficiency with single-skill triage algorithms among relatively uniform prehospital groups, tier 2 covered integrated decision-making involving multiple competencies within multidisciplinary clinical teams, and tier 3 included nonclinical stakeholders, focusing on system readiness, organizational coordination, and policy changes. These tiers are preliminary patterns derived inductively and should be validated with larger samples.

#### Tier 1: Algorithm Rehearsal

Two studies examined how individual responders interacted with a triage protocol by using TTXs to practice and assess specific procedures in controlled settings [25,26]. These exercises evaluated triage accuracy, used standardized scoring, and tested performance within short time frames. McGlynn et al [26] used patient groups of 10, 100, and 1000 casualties, while Cheng et al [25] compared 6 pediatric triage algorithms. Participants were mainly prehospital professionals trained in the relevant protocols. This phase focused on measuring individual accuracy and the correct application of algorithms rather than on team coordination or broader decision-making contexts.

#### Tier 2: Scenario-Based Decision Training

Seven studies focused on interaction within interdisciplinary teams, using TTXs to enhance multicompetency decision-making under realistic operational constraints [4,27-32]. These exercises went beyond triage to involve incident command, team coordination, resource management, CBRNE (Chemical, Biological, Radiological, Nuclear, Explosive) response, leadership, and ethical reasoning. Duration varied from 25 minutes to 2 days, with 1‐4 scenarios per session. Scenarios were tailored to specific contexts and included incidents such as a hydrofluoric acid chemical plant explosion [27]; fire, terrorism, and active shooter events [28]; mass shootings and fires [4]; 4 CBRNE situations [29]; a multivehicle accident [30]; triage across multiple disaster protocols [31]; and a building-collapse MCI scenario [32]. Participants were typically multidisciplinary, comprising prehospital personnel, emergency department staff, primary care providers, and sometimes public health officials. This tier prioritized integrated team decision-making under time constraints and uncertain information, rather than isolated skill assessment.

#### Tier 3: Systems Integration Testing

Four studies examined interorganizational and agency interactions through TTXs to evaluate coordination, collaboration, and policy response strategies [33-36]. These exercises engaged multiple agencies beyond clinical staff, such as hospital leaders, emergency services, firefighters, police, and other government entities. The scenarios included pediatric MCI management after a school bus crash [34], a CBRNE response with decontamination and triage [33], a combined suicide bombing and terrorist attack involving firearms [35], and postgraduate disaster medicine training focused on leadership and system coordination [36]. Unlike tiers 1 and 2, this level prioritized organizational preparedness, clarifying agency roles, formal after-action reviews, policy documentation, memoranda of understanding, and ongoing postexercise system improvements over immediate individual training.

### Facilitation Approaches Across Tiers

Across the 13 studies, 5 recurring facilitation modes were identified, each associated with particular tiers and learning goals. These modes reflected differences in the facilitator’s main role, such as guiding participants through a scenario, providing feedback on performance, ensuring consistent assessment, or supporting postexercise review. The following subsections describe the characteristics of each mode and its use across the 3 tiers.

#### Instructional Guide (Tier 2 Predominant)

Six tier 2 studies incorporated active instructional guidance in exercise scenarios [4,27,29-32]. Facilitators managed the scenario flow by providing timed information, posing clarifying questions, and offering prompts when decision-making slowed. They also adjusted scenario elements such as casualty numbers, resource availability, and time constraints to enhance learning. After each scenario, structured debriefings took place, often led by facilitators who asked questions to reinforce essential learning points.

#### Performance Coach (Tier 2)

The study by Phattharapornjaroen et al [28] involved placing 2 supervisory personnel in each exercise group, explicitly tasked with observing and giving iterative feedback on team leadership behaviors and decision-making quality, using a combination of live observation and structured feedback.

#### Standardization Agent (Tier 1 Predominant)

McGlynn et al [26] and Cheng et al [25] emphasized measurement consistency and the reliability of assessments by using standardized facilitation protocols [25,26]. Facilitators underwent rater calibration training, used reference sheets and standardized scoring guides, and followed scripted scenario presentations to ensure interrater reliability.

#### Expert-Led Facilitation (Tier 3)

Cicero et al [34] and Skryabina et al [35] involved senior clinicians and emergency preparedness specialists in conducting exercises and guiding after-action reports. Their role therefore extended beyond scenario delivery to postexercise review, consistent with tier 3’s focus on organizational preparedness and interagency coordination.

#### Multidisciplinary Facilitation (Tier 3)

Anan et al [33] and Zimmerman et al [36] employed multidisciplinary facilitation teams from various agencies to guarantee scenario realism and credibility within each participant’s operational setting. In both modes, tier 3 facilitation emphasized interagency collaboration and systems-level learning over individual skill evaluation. Figure 4 shows the alignment between TTX tiers and facilitation modes.

**Figure 4.**
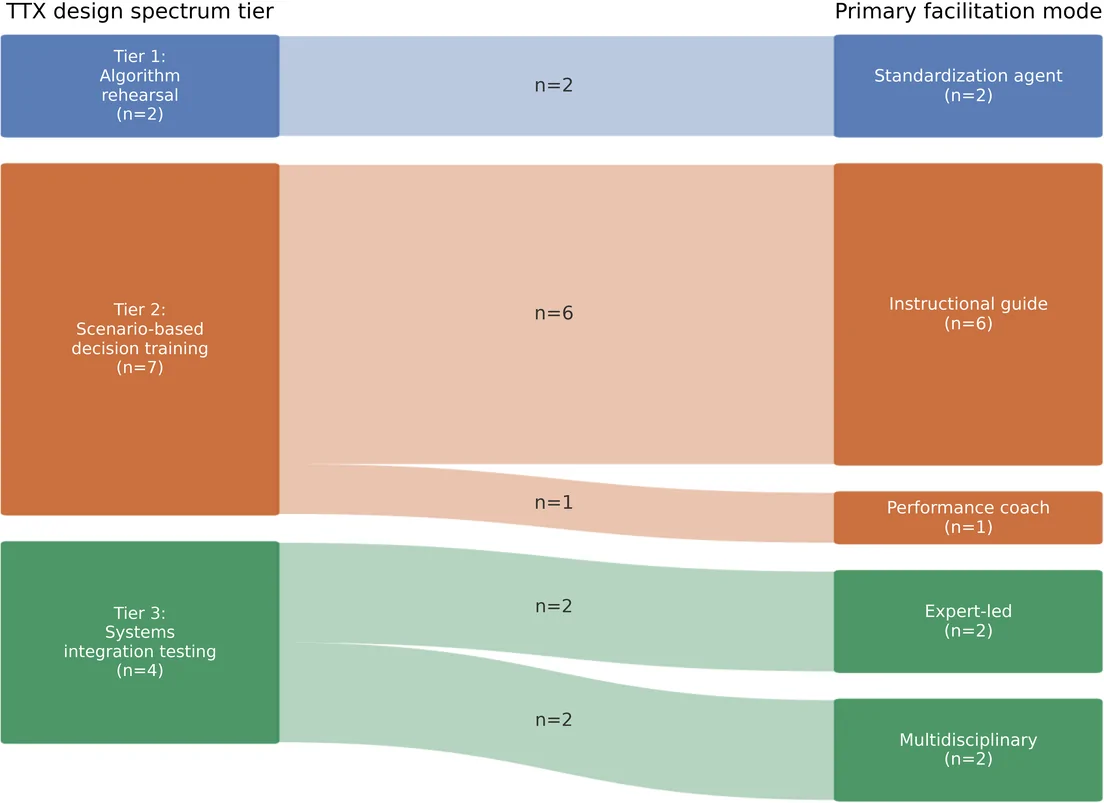
TTX tier–facilitation mode alignment (N=13). Alluvial diagram illustrating the connection between TTX Design Spectrum tiers (on the left) and primary facilitation modes (on the right). Blue represents tier 1 (algorithm rehearsal), brown represents tier 2 (scenario-based decision training), and green represents tier 3 (systems integration testing). The lighter-shaded flows retain the color of their source tier, and flow width corresponds to the number of studies. Each study is linked to 1 specific tier and 1 primary facilitation mode. TTX: tabletop exercise.

### Evaluation Strategies and Kirkpatrick Mapping

#### Data Collection Instruments and Timing

The 13 studies used diverse data collection methods. Tools for Kirkpatrick levels 1‐2 included knowledge tests (used in 8 studies), satisfaction ratings (7 studies), confidence assessments (5 studies), and self-reports on knowledge, competency, or preparedness (also 5 studies). Reaction measures extended to perceived usefulness, usability, and relevance ratings. Multimedia Appendix 4 details the specific instruments mapped to each outcome level [4,25-36]. For level 2+ (Applied Learning), assessment methods included scoring triage accuracy and speed against reference standards [25,26,31], structured observation to evaluate team behaviors, task performance, decision quality during exercises [27-29,36], and after-action reports assessed across sequential scenarios [33].

Level 3 (Behavior) evidence includes documented use of exercise-based skills in real operational contexts. Two studies reported this level of evidence. Skryabina et al [35] observed that participants used exercise-based knowledge and decision-making during the Manchester Arena bombing response. Zimmerman et al [36] described postprogram role application, with participants taking on roles such as departmental preparedness coordinator and simulation instructor-candidate, suggesting early signs of transferring practice beyond the exercise environment.

Level 4 data pertain to organizational change. Cicero et al [34] developed hospital preparedness metrics based on a 2-year postexercise follow-up. Skryabina et al [35] observed improvements in casualty distribution across the system and enhanced operational efficiency during the actual MCI response. Zimmerman et al [36] noted early organizational impacts, such as establishing a structured simulation platform, conducting additional course iterations, holding refresher meetings, engaging in alumni follow-up activities, and continuously refining contingency plans. Most studies used pre- and postassessment timings, with some also including during-exercise measurements such as triage accuracy and incident command compliance. Additionally, 1 study incorporated a 1-week delayed posttest to assess knowledge retention [31].

#### Kirkpatrick-Tier Mapping

Across the 13 studies, the distribution of Kirkpatrick levels was as follows: level 1 (Reaction) n=10, level 2 (Learning) n=10, level 2+ (Applied Learning) n=8, level 3 (Behavior) n=2, and level 4 (Results) n=3. The level 2+ category indicates a measurement pattern that the standard 4-level model does not clearly depict. The study-level distribution of Kirkpatrick outcome assessments across tiers is shown in Figure 3, with detailed outcome mapping by study available in Multimedia Appendix 4. Evaluation depth varied between tiers (Table 3). Outcomes for levels 3 and 4 were assessed solely in systems integration (tier 3) studies [34-36], aligning with this tier’s emphasis on systems-level readiness and real-world validation opportunities.

**Table 3.** Kirkpatrick outcome assessment by Tabletop Exercise Design Spectrum tier (number of studies assessing each level).^a^

Tier	Studies	Level 1: Reaction	Level 2: Learning	Level 2+ (Applied Learning)	Level 3: Behavior	Level 4: Results
1: algorithm rehearsal	2	1	0	2	0	0
2: scenario-based decision training	7	6	7	4	0	0
3: systems integration testing	4	3	3	2	2	3
Total (N=13)	13	10	10	8	2	3

a Cell values represent the number of studies evaluating each level; a single study can contribute to multiple levels. Level 2+ (Applied Learning) indicates structured performance assessments conducted within the exercise environment (Multimedia Appendix 2).

### Frameworks Guiding Exercise Design and Evaluation

#### Overview

Figure 5 provides a summary of how training design, assessment, and operational frameworks are reported across the 13 included studies. Operational frameworks were documented most consistently, while specific educational design and assessment frameworks appeared less frequently.

**Figure 5.**
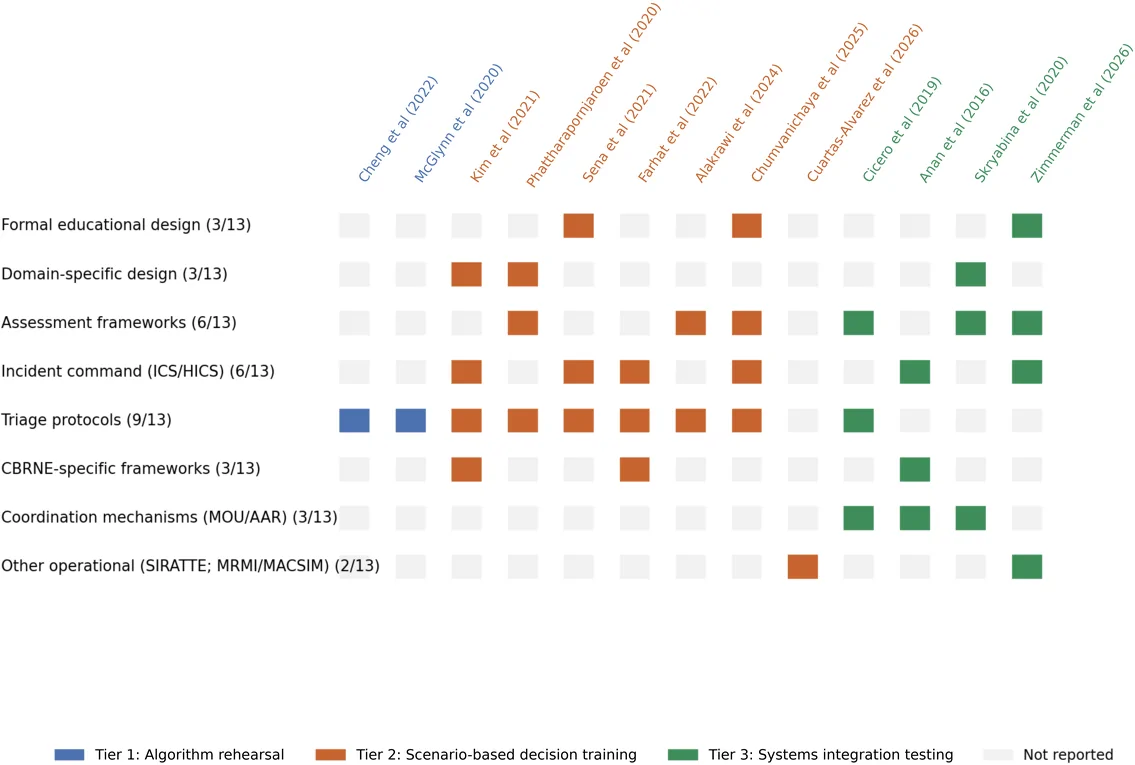
Framework reporting landscape across included studies (N=13). Matrix chart displaying framework reporting across studies, categorized into 8 groups: formal educational design, domain-specific design, assessment frameworks, and 5 operational areas: incident command (ICS/HICS), triage protocols, CBRNE-specific frameworks, coordination mechanisms (such as memoranda of understanding and after-action reports), and other operational frameworks (such as SIRATTE; MRMI/MACSIM). Columns represent individual studies, color-coded by TTX Design Spectrum tier; filled cells denote reported frameworks, while light gray cells show frameworks not reported [4,25-36]. AAR: after-action report; CBRNE: Chemical, Biological, Radiological, Nuclear, Explosive; HICS: Hospital Incident Command System; ICS: Incident Command System; MACSIM: Mass Casualty Simulation System; MOU: Memorandum of Understanding; MRMI: Medical Response to Major Incidents; SIRATTE: Security, Information, Roles, Areas, Triage, Treatment, and Evacuation.

#### Training Design Frameworks

Out of 13 studies, only 3 explicitly described formal educational training frameworks. Chumvanichaya et al [31] used backward design, focusing the exercise on previously identified preparedness gaps. Sena et al [4] applied Kolb’s [37] experiential learning model and adult learning principles to shape scenario development and debriefings. Zimmerman et al [36] integrated disaster medicine education and competency frameworks into postgraduate training. Seven studies did not specify any formal educational design framework [25,26,29,30,32-34]. Meanwhile, 3 studies used domain-specific frameworks instead of broad educational models: Kim et al [27] organized CBRNE response phases around the Chain of Chemical Survival, Phattharapornjaroen et al [28] applied the 3LC (3-Level Collaboration) framework with Major Incident Medical Management and Support or Medical Response to Major Incidents triage protocols, and Skryabina et al [35] based their approach on the Emergency Preparedness Cycle and Emergo Train System. Overall, most research relied more on disaster response or operational frameworks than on explicit educational design theories.

#### Assessment Frameworks

Assessment frameworks were described more clearly than training design frameworks. Phattharapornjaroen et al [28] used the CSCATTT (Command, Safety, Communication, Assessment, Triage, Treatment, Transportation) framework to organize and assess incident command performance. Cicero et al [34] used the Hospital Pediatric Disaster Preparedness Checklist and after-action reports to evaluate organizational readiness. Skryabina et al [35] integrated Kirkpatrick levels with the Promoting Action on Research Implementation in Health Services framework to analyze both training outcomes and implementation processes. Other studies used specific structured tools for assessments: Chumvanichaya et al [31] applied the Attention, Relevance, Confidence, Satisfaction Motivation Model to measure participant engagement, Alakrawi et al [30] adapted the Competency Level Use in Triage Scale to evaluate triage competency, and Zimmerman et al [36] used a 29-item questionnaire along with CSCATTT-anchored instructor scores to assess applied performance during simulations.

#### Operational Frameworks

Operational frameworks were reported more consistently than educational design frameworks. Six studies mentioned the Incident Command System or Hospital Incident Command System [4,27,29,31,33,36]. Triage protocols, such as Simple Triage and Rapid Treatment; Sort, Assess, Lifesaving Interventions, Treatment/Transport; Sieve (UK Major Incident Primary Triage); Secondary Triage (UK Major Incident); JumpSTART; CareFlight; Pediatric Triage Tape; and Sacco triage, were the most common operational frameworks used across different tiers [4,25-31,34]. Three studies used CBRNE-specific response frameworks: the Chain of Chemical Survival [27], CBRNE workshop competencies [29], and the MCLS-CBRNE course framework [33]. Tier 3 studies more frequently included coordination mechanisms such as memoranda of understanding, centralized dispatch protocols, interagency responsibilities, and after-action reports [33-35]. Cuartas-Alvarez et al [32] used SIRATTE, a prehospital MCI framework that covers Security, Information, Roles, Areas, Triage, Treatment, and Evacuation, in the MassCas tabletop game. Zimmerman et al [36] incorporated several operational and collaborative frameworks, such as Incident Command System, CSCATTT, 3LC, Medical Response to Major Incidents, and Mass Casualty Simulation System, into its postgraduate disaster medicine program.

## Discussion

### Principal Findings

The main conclusion of this scoping review is that TTXs in prehospital MCI preparedness should be viewed as a group of related exercise types, each serving different educational and operational goals, rather than a single, uniform intervention. These purposes are categorized into 3 tiers: algorithm rehearsal, scenario-based decision training, and systems integration testing. Outcomes are mapped using the Kirkpatrick framework, with level 2+ indicating structured performance during the exercise that lies conceptually between knowledge acquisition and real-world application. These tiers represent increasing levels of interaction: between individual responders and protocols, among interdisciplinary team members, and across organizations and agencies. Facilitation modes involve intentionally designed interactions between facilitators and participants. While diverse exercise designs are common and often suitable, the key message is that the purpose, facilitation, and evaluation of the exercise must be explicitly aligned. Without a shared typology of purpose, evidence from exercises with different goals cannot be meaningfully compared. As shown in Figure 6, the literature indicates a pattern where exercise purpose, facilitation mode, and evaluation depth progress together.

**Figure 6.**
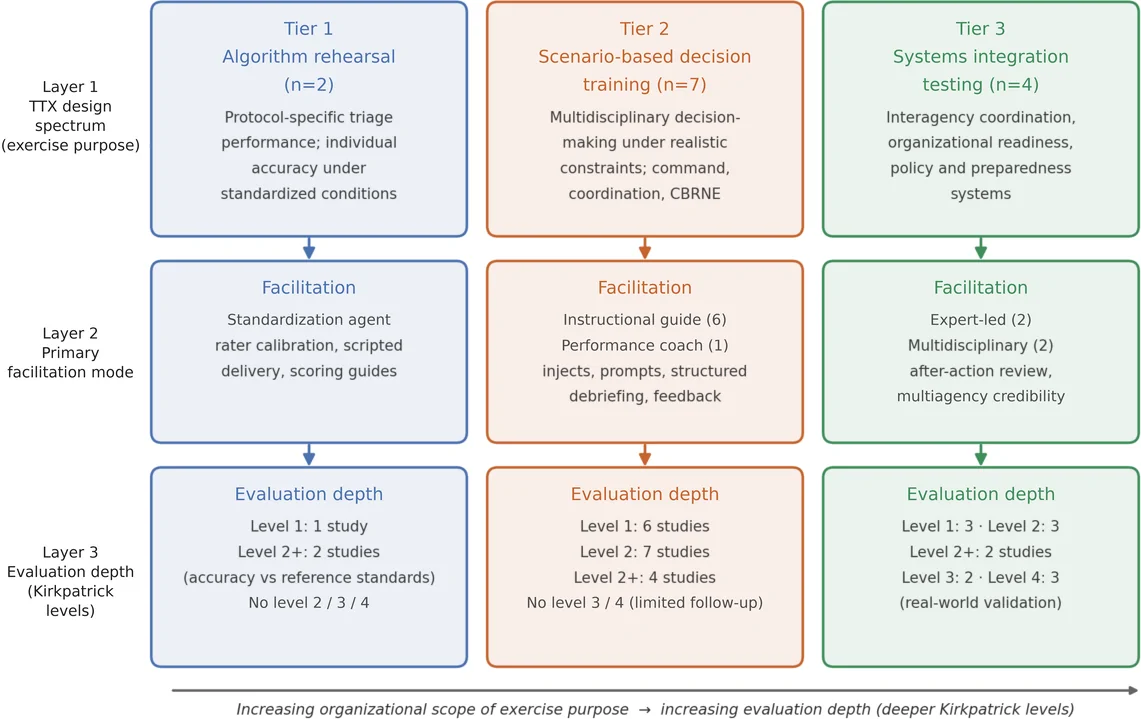
Integrated overview of TTX purpose, facilitation, and evaluation depth across included studies (N=13). The figure outlines the included studies across 3 interconnected layers: (1) the TTX Design Spectrum tier, (2) the main facilitation modes associated with each tier, and (3) the evaluation depth categorized by Kirkpatrick levels, including level 2+ (Applied Learning). CBRNE: Chemical, Biological, Radiological, Nuclear, Explosive; TTX: tabletop exercise.

The tier typology should be seen as an initial guideline rather than a confirmed classification. Nonetheless, it clarifies why studies labeled as TTXs often appeared quite different in practice: algorithm-rehearsal exercises focused on standardized practice of specific skills [25,26], scenario-based decision training involved integrated clinical and command decisions in discussion settings [4,27-32], and systems-integration exercises targeted organizational coordination and system readiness [33-36]. Exercises aimed at rehearsing an algorithm, enhancing team decision-making, or testing system coordination do not share the same facilitation approach or outcome metrics.

The Design Spectrum should be aligned with existing exercise doctrine. The HSEEP (Homeland Security Exercise and Evaluation Program) connects exercise goals to structured assessments using capability-based exercise evaluation guides and standardized after-action reports [38]. However, HSEEP treats TTXs as a single, uniform discussion-based type. The current typology offers a more detailed classification within that category, identifying different types of TTXs that require varied facilitation and evaluation approaches. This typology is designed to complement HSEEP objectives and not replace them.

This has a practical implication for anyone designing or assessing a TTX: the evaluation approach should be aligned with the exercise’s goal, not judged by a universal standard of assessment thoroughness. A focused protocol rehearsal may rightly prioritize structured performance metrics such as decision accuracy and interrater reliability, without necessarily claiming to measure organizational impact [25,26]. Meanwhile, a decision-training exercise might aim to improve knowledge, confidence, communication, and role clarity but should ideally incorporate follow-up methods if it seeks to influence future practice [4,29,32]. A systems-centered exercise should analyze after-action processes, coordination structures, and readiness outputs, as these are its core focus [34-36]. The key point is not whether every TTX achieves the highest Kirkpatrick level but whether the chosen level is appropriate for the capability being tested.

### Separating Applied Learning From Behavioral Transfer

The level 2+ subcategory is valuable because it differentiates between applied performance during the exercise and behavioral transfer outside of it. Multiple studies have assessed applied performance during the tabletop, providing evidence of learning in a simulated environment [25-29,31,33,36]. While this evidence is educationally significant, it does not necessarily reflect practice change in real settings. Making this distinction helps prevent overinterpreting simulation results as proof of real-world transfer. This differentiation is not new; models such as the revised Kirkpatrick framework, Miller’s framework, and translational science models of simulation-based education outcomes all recognize it [22-24]. However, its systematic application to prehospital MCI tabletop evidence has not been reported before. Broader reviews have generally shown clustering at the Reaction and Learning levels without separating structured in-exercise performance from actual operational transfer [7,8].

This distinction also assists in understanding the limited occurrence of specific behaviors and outcome results. Their absence should not be automatically interpreted as failure. In prehospital disaster education, actual events are rare, unpredictable, and difficult to tie to a single training session [39], making it challenging to observe behavioral transfer even when learning has occurred. Additionally, follow-up methods in scenario-based decision-training studies were often limited [4,29,30,32], with only a single delayed posttest after 1 week [31], which hampers the ability to determine whether these exercises impacted later operational reasoning. This does not imply that such exercises are ineffective but rather that they lacked sufficient follow-up relative to their expected outcomes, highlighting a gap in evaluation data rather than a flaw in design.

### Timing, Transfer, and Facilitation

The study by Skryabina et al [35] provides rare naturalistic evidence that exercise learning can be applied in real responses when training is close to deployment. It found that participants reported using knowledge from the TTX during the Manchester Arena incident, which occurred soon afterward. Although this single uncontrolled observation cannot establish causality, it aligns with the literature on distributed practice, which shows that the spacing and timing of learning opportunities influence retention and transfer [40], and with the advice by Cicero et al [34] to exercise every 6 months [34]. While single-exposure TTXs may support learning, lasting behavior change likely depends more on repetition and deliberate timing than on a one-time exposure. This remains an open question for future research rather than a definitive conclusion of this review.

Facilitation findings consistently indicate that it is a strategic design choice rather than a neutral delivery method, and that it varies with the exercise’s purpose (Figure 4). More directive, standardized facilitation is better suited for narrowly focused rehearsal activities, while expert-led, coaching, or multidisciplinary facilitation is typically used in broader scenario-based and system-level exercises [4,27,34,36]. There is no single facilitation style that is universally superior; instead, the facilitator’s role should be aligned with the exercise’s goals, whether to standardize performance, encourage collaborative reasoning, identify coordination gaps, or foster organizational reflection.

### Frameworks and Implementation

Operational frameworks were described more consistently than educational or assessment frameworks (Figure 5). Only a few studies explicitly detailed formal educational frameworks such as experiential learning, backward design, or competency-based disaster medicine education [4,31,36]. While the field is grounded in operational principles, its pedagogical aspects are less well articulated. Operational doctrine can effectively shape scenarios, but inconsistent reporting of educational and assessment approaches hampers reproducibility and obscures which design choices actually enhance learning or transfer. Notably, the combination of the Kirkpatrick model with the Promoting Action on Research Implementation in Health Services implementation framework by Skryabina et al [35] stands out, as it connects training outcomes with implementation processes rather than viewing evaluation as an isolated end point [35,41,42]. This approach demonstrates how exercise evaluation can more systematically contribute to system-level preparedness improvements.

These findings generally align with earlier review literature but offer a more specific interpretive perspective. Frégeau et al [7] observed outcomes primarily at lower Kirkpatrick levels in medical emergency contexts, while Emaliyawati et al [8] noted that tabletop disaster exercises enhance knowledge, confidence, preparedness, and performance. This review differs by focusing on the prehospital MCI interface, introducing the level 2+ category, and suggesting that the main challenge is purpose-aligned evaluation rather than measurement deficiencies. This distinction is practically valuable, helping educators and planners determine the kind of evidence they can reasonably expect from various exercises.

The clearest takeaway from practice is that TTXs should be designed starting from the desired capability. If the aim is to rehearse algorithms, then structured in-exercise performance measures might suffice as end points. For scenario-based decision training, evaluation should go beyond participant satisfaction and include follow-up methods such as delayed case-based testing, repeated scenarios, structured interviews, or local practice audits. If the focus is on systems integration, exercises should connect to after-action reviews, policy updates, coordination metrics, or preparedness planning [34-36]. This review advocates for a proportional evaluation approach: not every exercise needs maximum evaluation depth, but each should have an evaluation strategy aligned with its purpose. Multimedia Appendix 5 summarizes these pairings, listing typical objectives, facilitation modes, evaluation targets, example instruments from the studies, and suggested data to record [25-28,31,33-36].

### Future Research

Future research should focus on refining this purpose alignment approach instead of merely questioning whether TTXs “work” in general. One key area is to develop practical methods for measuring transfer following scenario-based decision-training exercises without relying on the occurrence of a real MCI: options include repeating scenarios, using follow-up decision vignettes, conducting interval audits of triage or command processes, and linking short-format exercises as possible next steps. Another important area is to investigate whether repeated or ongoing exposure to related TTXs enhances retention and transfer more than isolated sessions. This is especially relevant since the current review does not establish a dose-response relationship; however, the training-transfer literature suggests that this is plausible because transfer relies on reinforcement, opportunities for application, and workplace conditions [43,44].

A third priority involves stakeholder-based implementation research, engaging EMS leaders, emergency preparedness officers, public health partners, hospital interface stakeholders, and simulation faculty in designing exercise objectives and follow-up plans. This ensures that evaluations align with real operational needs. It should also include a more structured, comparable collection of evaluation data, such as observation ratings, accuracy scores, and after-action findings, using existing templates such as HSEEP’s Exercise Evaluation Guides and standardized reporting [38]. A fourth priority is expanding scenarios beyond specific, bounded incidents. Predictable hazards such as storms, wildfires, floods, or hurricanes could be especially valuable, as threat windows can sometimes be forecasted. Advances in satellite hazard assessments, artificial intelligence–enabled risk estimation, and forecasting may help identify these periods and enable more realistic timing of training relative to operational demand [39,45]. These designs could also examine whether training closer to deployment improves transfer.

### Limitations

Several limitations should inform how these findings are interpreted. First, the evidence base was limited in size and diverse in nature, so the proposed tiers and alignment patterns should be viewed as preliminary, hypothesis-generating concepts rather than confirmed frameworks. Specifically, since the tier definitions partly depended on participant composition and outcome scope, the link between tier and evaluation depth is partly inherent and should not be seen as an independent empirical correlation. Second, this was a scoping review without formal critical appraisal; thus, the findings depict the state of the literature rather than comparing the effectiveness of different TTX designs. Most studies included used pre-post, quasi-experimental, descriptive, or mixed methods approaches, which restrict causal conclusions.

Third, the study reporting was inconsistent. The definitions of outcomes, facilitation descriptions, and framework reporting varied, leading to interpretive uncertainty when aligning studies across tiers and Kirkpatrick levels. The level 2+ subcategory enhances conceptual clarity, but its assignment still relied on reviewer judgment. Fourth, the literature was mainly from high-income or upper-middle-income settings and focused on human-caused or bounded hazard scenarios, restricting its applicability to resource-limited environments and prolonged natural disasters (Figure 2). Additionally, limiting the review to English-language sources might have excluded important evidence.

Two of the review’s more speculative points rely on limited evidence. The idea that temporal proximity between training and real events might facilitate transfer primarily derives from a single naturalistic case [35]. While interesting, it remains unconfirmed. Similarly, the notion that short, time-limited tabletop formats may lack the functional fidelity, according to the definition by Hamstra et al [46], to simulate long-term, evolving disasters that disrupt infrastructure is speculative. The existing literature primarily involves bounded incidents and does not directly test this idea. Both points are presented as suggestions for future research rather than confirmed explanations.

### Conclusions

TTXs for prehospital MCI preparedness should not be seen as a single, uniform approach. Their success mainly depends on how clearly the designers define the expected level of interaction. Whether it is an individual following protocols, a multidisciplinary team working together, or organizations coordinating efforts, facilitation and evaluation should be tailored accordingly. By concentrating on the prehospital MCI literature and differentiating between applied learning during exercises (level 2+) and actual behavioral transfer, while considering variations through the lens of design–evaluation alignment, this review offers a targeted synthesis of the prehospital MCI interface that complements earlier, broader tabletop reviews [7,8]. When used carefully, TTXs can remain a scalable and strategic component of prehospital disaster preparedness, with further research needed to determine when and how they promote lasting practice change and system readiness.

## Supplementary material

10.2196/96228 Multimedia Appendix 1 Complete database-specific search strategies.

10.2196/96228 Multimedia Appendix 2 Glossary, Kirkpatrick classification rules, and Tabletop Exercise Design Spectrum definitions.

10.2196/96228 Multimedia Appendix 3 Metadata for unretrieved conference abstracts.

10.2196/96228 Multimedia Appendix 4 Kirkpatrick outcome mapping by study and exercise tier.

10.2196/96228 Multimedia Appendix 5 Practitioner summary: purpose-aligned design, facilitation, and evaluation by tabletop exercise tier.

10.2196/96228 Checklist 1 PRISMA-ScR checklist.

## Abbreviations

CBRNE: Chemical, Biological, Radiological, Nuclear, Explosive
CSCATTT: Command, Safety, Communication, Assessment, Triage, Treatment, Transportation
EMS: emergency medical services
HSEEP: Homeland Security Exercise and Evaluation Program
MCI: mass casualty incident
PRISMA: Preferred Reporting Items for Systematic Reviews and Meta-Analyses
PRISMA-S: PRISMA Extension for Reporting Literature Searches
PRISMA-ScR: Preferred Reporting Items for Systematic Reviews and Meta-Analyses Extension for Scoping Reviews
3LC: 3-Level Collaboration
TTX: tabletop exercise
