# Supplementary material for: Design, Facilitation, and Evaluation of Tabletop Exercises for Prehospital Mass Casualty Preparedness: Scoping Review
Source: Interact J Med Res. 2026 Oct 5;15:e96228. doi: 10.2196/96228 (PMC13637994; doi:10.2196/96228)
Supplement: Supplementary file 1 [file ijmr-v15-e96228-s001.docx]

Multimedia Appendix 1. Search Strategy

| Database | Search strategy | Res |
| --- | --- | --- |
| PubMed | (( (((((((((((((((((((((((((((((paramedic*) OR (first responder)) OR (paramedics)) OR (paramedic)) OR (first responders)) OR (first responder*)) OR (ambulance*)) OR (ambulance personnel)) OR (ambulance staff)) OR (emergency medical service*)) OR (EMS)) OR (EMT)) OR (EMTs)) OR (emergency medical technician*)) OR (prehospital)) OR (pre-hospital)) OR (prehospital provider*)) OR (prehospital personnel)) OR (out-of-hospital)) OR (emergency responder*)) OR (rescue personnel)) OR (emergency physician*)) OR (emergency medicine resident*)) OR (nurse*)) OR (physician*)) OR (public health officer*)) OR (emergency manager*)) OR (emergency planner*)) OR (medical student*)) OR (medical students)) AND ( (((((((((((((((((((((((((((((((((tabletop exercises) OR (table-top exercise)) OR (table-top exercises)) OR (table top exercise)) OR (table top exercises)) OR (tabletop simulation)) OR (tabletop simulations)) OR (table-top simulation)) OR (table-top simulations)) OR (table top simulation)) OR (table top simulations)) OR (TTX)) OR (TTXs)) OR (tabletop)) OR (tabletop drill)) OR (tabletop drills)) OR (discussion-based exercise)) OR (discussion-based exercises)) OR (discussion based exercise)) OR (discussion based exercises)) OR (discussion-based simulation)) OR (discussion based simulation)) OR (scenario-based exercise)) OR (scenario-based exercises)) OR (scenario based exercise)) OR (scenario based exercises)) OR (scenario-based simulation)) OR (scenario-based simulations)) OR (scenario based simulations)) OR (paper-based exercise)) OR (paper based exercise)) OR (game-based simulation)) OR (gamified exercise)) OR (gamified simulation))) AND (((((((( ((((((((((((((((((((((((((((((((emergency preparedness) OR (disaster preparedness)) OR (disaster planning)) OR (emergency planning)) OR (emergency response)) OR (disaster response)) OR (mass casualty incident*)) OR (mass casualty event*)) OR (mass casualty response)) OR (mass casualty management)) OR (MCI)) OR (MCIs)) OR (multiple casualty incident*)) OR (major incident*)) OR (major incident response)) OR (field triage)) OR (triage)) OR (incident command)) OR (incident command system)) OR (ICS)) OR (scene command)) OR (scene coordination)) OR (prehospital care)) OR (pre-hospital care)) OR (disaster simulation)) OR (response coordination)) OR (resource allocation)) OR (CBRN)) OR (CBRNE)) OR (chemical incident)) OR (biological incident)) OR (radiological incident)) OR (nuclear incident)) OR (hazardous materials)) OR (HAZMAT)) OR (terrorism)) OR (bombing)) OR (explosion)) OR (active shooter)) OR (mass shooting)) | 1,066 |
| Scopus | TITLE-ABS-KEY ( paramedic* OR "first responder*" OR ambulance* OR "ambulance personnel" OR "ambulance staff" OR "emergency medical service*" OR EMS OR EMT OR EMTs OR "emergency medical technician*" OR prehospital OR "pre-hospital" OR "prehospital provider*" OR "prehospital personnel" OR "out-of-hospital" OR "out of hospital" OR "emergency responder*" OR "rescue personnel" OR "emergency physician*" OR "emergency medicine resident*" OR nurse* OR physician* OR "public health officer*" OR "emergency manager*" OR "emergency planner*" OR "medical student*" ) AND TITLE-ABS-KEY ( "tabletop exercise*" OR "table-top exercise*" OR "table top exercise*" OR "tabletop simulation*" OR "table-top simulation*" OR "table top simulation*" OR TTX OR TTXs OR tabletop OR "tabletop drill*" OR "discussion-based exercise*" OR "discussion based exercise*" OR "discussion-based simulation*" OR "discussion based simulation*" OR "scenario-based exercise*" OR "scenario based exercise*" OR "scenario-based simulation*" OR "scenario based simulation*" OR "paper-based exercise" OR "paper based exercise" OR "game-based simulation" OR "gamified exercise" OR "gamified simulation" ) AND TITLE-ABS-KEY ( "emergency preparedness" OR "disaster preparedness" OR "disaster planning" OR "emergency planning" OR "emergency response" OR "disaster response" OR "mass casualty incident*" OR "mass casualty event*" OR "mass casualty response" OR "mass casualty management" OR MCI OR MCIs OR "multiple casualty incident*" OR "major incident*" OR "major incident response" OR "field triage" OR triage OR "incident command" OR "incident command system" OR ICS OR "scene command" OR "scene coordination" OR "prehospital care" OR "pre-hospital care" OR "disaster simulation" OR "response coordination" OR "resource allocation" OR CBRN OR CBRNE OR "chemical incident" OR "biological incident" OR "radiological incident" OR "nuclear incident" OR "hazardous materials" OR HAZMAT OR terrorism OR bombing OR explosion OR "active shooter" OR "mass shooting" ) | 156 |
| Embase | #81 #18 AND #42 AND #80  #80 #43 OR #44 OR #45 OR #46 OR #47 OR #48 OR #49 OR #50 OR #51 OR #52 OR #53 OR #54 OR #55 OR #56 OR #57 OR #58 OR #59 OR #60 OR #61 OR #62 OR #63 OR #64 OR #65 OR #66 OR #67 OR #68 OR #69 OR #70 OR #71 OR #72 OR #73 OR #74 OR #75 OR #76 OR #77 OR #78 OR #79  #79 'mass shooting'  #78 'explosion'  #77 'bombing'  #76 'terrorism'  #75 'nuclear incident'  #74 'biological incident'  #73 'radiological incident'  #72 'chemical incident'  #71 'cbrne'  #70 'cbrn'  #69 'casualty distribution'  #68 'response coordination'  #67 'pre-hospital care'  #66 'emergency care'  #65 'scene coordination'  #64 'scene command'  #63 'command and control'  #62 'ics'  #61 'incident command system'  #60 'incident command'  #59 'patient triage'  #58 'field triage'  #57 'major incident response'  #56 'major incident'  #55 'multiple casualty incident'  #54 'mcis'  #53 'mci'  #52 'mass casualty management'  #51 'mass casualty response'  #50 'mass casualty event'  #49 'mass disaster'  #48 'disaster response'  #47 'emergency response'  #46 'emergency planning'  #45 'disaster planning'  #44 'disaster preparedness'  #43 'emergency preparedness'  #42 #19 OR #20 OR #21 OR #22 OR #23 OR #24 OR #25 OR #26 OR #27 OR #28 OR #29 OR #30 OR #31 OR #32 OR #33 OR #34 OR #35 OR #36 OR #37 OR #38 OR #39 OR #40 OR #41  #41 'discussion based exercise'  #40 'discussion-based simulation'  #39 'preparedness exercise'  #38 'simulation exercises'  #37 'scenario-based exercises'  #36 'scenario based simulations'  #35 'paper based exercise'  #34 'simulation game'  #33 'simulation games'  #32 'game-based simulation'  #31 'gamified exercise'  #30 'gamified simulation'  #29 'tabletop drill'  #28 'ttx'  #27 'table top simulation'  #26 'table-top simulations'  #25 'table-top simulation'  #24 'tabletop simulations'  #23 'tabletop simulation'  #22 'table top exercise'  #21 'table-top exercises'  #20 'table-top exercise'  #19 'tabletop exercises'  #18 #1 OR #2 OR #3 OR #4 OR #5 OR #6 OR #7 OR #8 OR #9 OR #10 OR #11 OR #12 OR #13 OR #14 OR #15 OR #16 OR #17  #17 'physician'  #16 'nurse'  #15 'emergency medicine resident'  #14 'emergency physician'  #13 'out of hospital'  #12 'prehospital personnel'  #11 'prehospital provider'  #10 'pre-hospital'  #9 'prehospital'  #8 'emergency care'  #7 'emergency medical technician'  #6 'emergency health service'  #5 'rescue personnel'  #4 'ambulance personnel'  #3 'ambulance'  #2 'first responder (person)'  #1 'paramedical personnel'/exp OR 'paramedical personnel' | 185 |
| PsycNE T (PsycINFO) | Any Field: paramedic* OR Any Field: "first responder*" OR Any Field: ambulance* OR Any Field: "ambulance personnel" OR Any Field: "ambulance staff" OR Any Field: "emergency medical service*" OR Any Field: EMS OR Any Field: EMT OR Any Field: EMTs OR Any Field: "emergency medical technician*" OR Any Field: prehospital OR Any Field: "pre-hospital" OR Any Field: "prehospital provider*" OR Any Field: "prehospital personnel" OR Any Field: "out-of-hospital" OR Any Field: "out of hospital" OR Any Field: "emergency responder*" OR Any Field: "rescue personnel" OR Any Field: "emergency physician*" OR Any Field: "emergency medicine resident*" OR Any Field: nurse* OR Any Field: physician* OR Any Field: "public health officer*" OR Any Field: "emergency manager*" OR Any Field: "emergency planner*" OR Any Field: "medical student*" AND Any Field: "tabletop exercise*" OR Any Field: "table-top exercise*" OR Any Field: "table top exercise*" OR Any Field: "tabletop simulation*" OR Any Field: "table-top simulation*" OR Any Field: "table top simulation*" OR Any Field: TTX OR Any Field: TTXs OR Any Field: tabletop OR Any Field: "tabletop drill*" OR Any Field: "discussion-based exercise*" OR Any Field: "discussion based exercise*" OR Any Field: "discussion-based simulation*" OR Any Field: "discussion based simulation*" OR Any Field: "scenario-based exercise*" OR Any Field: "scenario based exercise*" OR Any Field: "scenario-based simulation*" OR Any Field: "scenario based simulation*" OR Any Field: "paper-based exercise" OR Any Field: "paper based exercise" OR Any Field: "game-based simulation" OR Any Field: "gamified exercise" OR Any Field: "gamified simulation" AND Any Field: "emergency preparedness" OR Any Field: "disaster preparedness" OR Any Field: "disaster planning" OR Any Field: "emergency planning" OR Any Field: "emergency response" OR Any Field: "disaster response" OR Any Field: "mass casualty incident*" OR Any Field: "mass casualty event*" OR Any Field: "mass casualty response" OR Any Field: "mass casualty management" OR Any Field: MCI OR Any Field: MCIs OR Any Field: "multiple casualty incident*" OR Any Field: "major incident*" OR Any Field: "major incident response" OR Any Field: "field triage" OR Any Field: triage OR Any Field: "incident command" OR Any Field: "incident command system" OR Any Field: ICS OR Any Field: "scene command" OR Any Field: "scene coordination" OR Any Field: "prehospital care" OR Any Field: "pre-hospital care" OR Any Field: "disaster simulation" OR Any Field: "response coordination" OR Any Field: "resource allocation" OR Any Field: CBRN OR Any Field: CBRNE OR Any Field: "chemical incident" OR Any Field: "biological incident" OR Any Field: "radiological incident" OR Any Field: "nuclear incident" OR Any Field: "hazardous materials" OR Any Field: HAZMAT OR Any Field: terrorism OR Any Field: bombing OR Any Field: explosion OR Any Field: "active shooter" OR Any Field: "mass shooting" | 11 |
| CINAHL | (paramedic* OR "first responder*" OR ambulance* OR "ambulance personnel" OR "ambulance staff" OR "emergency medical service*" OR EMS OR EMT OR EMTs OR "emergency medical technician*" OR prehospital OR "pre-hospital" OR "prehospital provider*" OR "prehospital personnel" OR "out-of-hospital" OR "out of hospital" OR "emergency responder*" OR "rescue personnel" OR "emergency physician*" OR "emergency medicine resident*" OR nurse* OR physician* OR "public health officer*" OR "emergency manager*" OR "emergency planner*" OR "medical student*") AND ("tabletop exercise*" OR "table-top exercise*" OR "table top exercise*" OR "tabletop simulation*" OR "table-top simulation*" OR "table top simulation*" OR TTX OR TTXs OR tabletop OR "tabletop drill*" OR "discussion-based exercise*" OR "discussion based exercise*" OR "discussion-based simulation*" OR "discussion based simulation*" OR "scenario-based exercise*" OR "scenario based exercise*" OR "scenario-based simulation*" OR "scenario based simulation*" OR "paper-based exercise" OR "paper based exercise" OR "game-based simulation" OR "gamified exercise" OR "gamified simulation") AND ("emergency preparedness" OR "disaster preparedness" OR "disaster planning" OR "emergency planning" OR "emergency response" OR "disaster response" OR "mass casualty incident*" OR "mass casualty event*" OR "mass casualty response" OR "mass casualty management" OR MCI OR MCIs OR "multiple casualty incident*" OR "major incident*" OR "major incident response" OR "field triage" OR triage OR "incident command" OR "incident command system" OR ICS OR "scene command" OR "scene coordination" OR "prehospital care" OR "pre-hospital care" OR "disaster simulation" OR "response coordination" OR "resource allocation" OR CBRN OR CBRNE OR "chemical incident" OR "biological incident" OR "radiological incident" OR "nuclear incident" OR "hazardous materials" OR terrorism OR bombing OR explosion OR "active shooter" OR "mass shooting") | 1260 |
| Cochra ne (CENTRAL) | paramedic* OR (first NEXT responder*) OR ambulance* OR (ambulance NEXT personnel) OR (ambulance NEXT staff) OR ((emergency NEXT medical) NEXT service*) OR EMS OR EMT OR EMTs OR ((emergency NEXT medical) NEXT technician*) OR prehospital OR pre-hospital OR (prehospital NEXT provider*) OR (prehospital NEXT personnel) OR out-of-hospital OR ((out NEXT of) NEXT hospital) OR (emergency NEXT responder*) OR (rescue NEXT personnel) OR (emergency NEXT physician*) OR ((emergency NEXT medicine) NEXT resident*) OR nurse* OR physician* OR ((public NEXT health) NEXT officer*) OR (emergency NEXT manager*) OR (emergency NEXT planner*) OR (medical NEXT student*) in Title Abstract Keyword AND (tabletop NEXT exercise*) OR ((table NEXT top) NEXT exercise*) OR (tabletop NEXT simulation*) OR ((table NEXT top) NEXT simulation*) OR TTX OR TTXs OR tabletop OR (tabletop NEXT drill*) OR ((discussion NEXT based) NEXT exercise*) OR ((discussion NEXT based) NEXT simulation*) OR ((scenario NEXT based) NEXT exercise*) OR ((scenario NEXT based) NEXT simulation*) OR ((paper NEXT based) NEXT exercise*) OR ((game NEXT based) NEXT simulation*) OR (gamified NEXT exercise) OR (gamified NEXT simulation) in Title Abstract Keyword AND (emergency NEXT preparedness) OR (disaster NEXT preparedness) OR (disaster NEXT planning) OR (emergency NEXT planning) OR (emergency NEXT response) OR (disaster NEXT response) OR ((mass NEXT casualty) NEXT incident*) OR ((mass NEXT casualty) NEXT event*) OR ((mass NEXT casualty) NEXT response) OR ((mass NEXT casualty) NEXT management) OR MCI OR MCIs OR ((multiple NEXT casualty) NEXT incident*) OR (major NEXT incident*) OR ((major NEXT incident) NEXT response) OR (field NEXT triage) OR triage OR (incident NEXT command) OR ((incident NEXT command) NEXT system) OR ICS OR (scene NEXT command) OR (scene NEXT coordination) OR (prehospital NEXT care) OR ((pre NEXT hospital) NEXT care) OR (disaster NEXT simulation) OR (response NEXT coordination) OR (resource NEXT allocation) OR CBRN OR CBRNE OR (chemical NEXT incident) OR (biological NEXT incident) OR (radiological NEXT incident) OR (nuclear NEXT incident) OR (hazardous NEXT material*) OR terrorism OR bombing OR explosion OR (active NEXT shooter) OR (mass NEXT shooting) in Title Abstract Keyword - (Word variations have been searched) | 14 |
| Clinic altrials.gov | (paramedic OR "first responder" OR ambulance OR "ambulance personnel" OR "ambulance staff" OR "emergency medical service" OR EMS OR EMT OR EMTs OR "emergency medical technician" OR prehospital OR "pre-hospital" OR "prehospital provider" OR "prehospital personnel" OR "out-of-hospital" OR "out of hospital" OR "emergency responder" OR "rescue personnel" OR "emergency physician" OR "emergency medicine resident" OR nurse OR physician OR "public health officer" OR "emergency manager" OR "emergency planner" OR "medical student") AND ("tabletop exercise" OR "table-top exercise" OR "table top exercise" OR "tabletop simulation" OR "table-top simulation" OR "table top simulation" OR TTX OR TTXs OR tabletop OR "tabletop drill" OR "discussion-based exercise" OR "discussion based exercise" OR "discussion-based simulation" OR "discussion based simulation" OR "scenario-based exercise" OR "scenario based exercise" OR "scenario-based simulation" OR "scenario based simulation" OR "paper-based exercise" OR "paper based exercise" OR "game-based simulation" OR "gamified exercise" OR "gamified simulation") AND ("emergency preparedness" OR "disaster preparedness" OR "disaster planning" OR "emergency planning" OR "emergency response" OR "disaster response" OR "mass casualty incident" OR "mass casualty event" OR "mass casualty response" OR "mass casualty management" OR MCI OR MCIs OR "multiple casualty incident" OR "major incident" OR "major incident response" OR "field triage" OR triage OR "incident command" OR "incident command system" OR ICS OR "scene command" OR "scene coordination" OR "prehospital care" OR "pre-hospital care" OR "disaster simulation" OR "response coordination" OR "resource allocation" OR CBRN OR CBRNE OR "chemical incident" OR "biological incident" OR "radiological incident" OR "nuclear incident" OR "hazardous materials" OR terrorism OR bombing OR explosion OR "active shooter" OR "mass shooting") | 1 |
