# Supplementary material for: Design, Facilitation, and Evaluation of Tabletop Exercises for Prehospital Mass Casualty Preparedness: Scoping Review
Source: Interact J Med Res. 2026 Oct 5;15:e96228. doi: 10.2196/96228 (PMC13637994; doi:10.2196/96228)
Supplement: Supplementary file 2 [file ijmr-v15-e96228-s002.docx]

# Multimedia Appendix 2. Glossary, Kirkpatrick Classification Rules, and TTX Design Spectrum Definitions

## Section 1. Kirkpatrick outcome classification

Level 1: Reaction. Participants’ immediate responses to the intervention, including satisfaction, perceived usefulness, engagement, relevance, and usability. Confidence ratings collected as immediate reactions or as motivation subscales (e.g., ARCS) were also classified here.

Level 2: Learning. Knowledge acquisition, skill development, or attitudinal change measured through quizzes, tests, checklists, validated questionnaires, or self-reported learning measures. Task-specific confidence (self-efficacy) measured before and after training as a learning outcome was classified as Level 2, consistent with the revised (New World) Kirkpatrick model (Kirkpatrick JD, Kirkpatrick WK. Kirkpatrick’s Four Levels of Training Evaluation. ATD Press; 2016).

Level 2+: Applied Learning within the exercise environment. Structured performance assessment conducted during or immediately within the tabletop or simulation environment. Outcomes were classified as Level 2+ when: (1) assessment occurred during or within the exercise scenario; (2) performance was evaluated against an external standard, rubric, observation protocol, or scoring system rather than self-report alone; and (3) the task required applied decision-making rather than simple recall.

Level 3: Behavior. Application of exercise-derived knowledge, skills, or decision-making patterns in real operational settings or post-exercise practice changes documented outside the exercise environment.

Level 4: Results. Organizational or system-level impact, such as adoption of new protocols, formalized interagency agreements, improved preparedness metrics, system-wide response improvement, or documented institutional change.

## Section 2. Kirkpatrick classification decision rules

1. Satisfaction, perceived usefulness, relevance, usability, and reaction-level or motivation-subscale confidence ratings were classified as Level 1.
2. Knowledge tests, quizzes, attitude scales, self-reported learning measures, and pre/post task-specific confidence (self-efficacy) measures were classified as Level 2. Post-only or unanchored confidence ratings defaulted to Level 1 unless they were explicitly measured as task-specific self-efficacy against the trained content.
3. Performance assessed during the exercise against a rubric, scoring guide, observer checklist, reference standard, or structured assessment protocol was classified as Level 2+.
4. Simulated performance was not classified as Level 3. Level 3 required evidence of behavior change or application outside the exercise environment.
5. Level 4 required organizational, institutional, or system-level impact documented through follow-up, policy change, preparedness metrics, or operational records.
6. When a study reported outcomes at multiple levels, each outcome was classified separately.

## Section 3. TTX Design Spectrum definitions

TTX Design Spectrum. This is a preliminary three-tier typology outlining the main purpose and scope of a tabletop exercise: Algorithm Rehearsal, Scenario-Based Decision Training, and Systems Integration Testing. It expands on the Homeland Security Exercise and Evaluation Program (HSEEP) typology by further differentiating within the tabletop exercise category, focusing on cognitive and organizational scope. Developed inductively through synthesis, it serves as an organizing typology rather than a validated framework.

- Tier 1: Algorithm Rehearsal. Exercises that concentrate on practicing or evaluating a specific protocol or algorithm, typically triage, with standardized scoring and individual performance evaluation.
- Tier 2: Scenario-Based Decision Training. Exercises designed to develop multiple competencies in realistic scenarios, covering triage, communication, coordination, resource allocation, incident command, and team decision-making.
- Tier 3: Systems Integration Testing. Exercises that concentrate on organizational, interagency, or system-level readiness, covering coordination among institutions, policy decision-making, memoranda of understanding, after-action reviews, and planning for preparedness.

## Section 4. Facilitation mode definitions (descriptive categories)

The terms below refer to common facilitation techniques seen in the included studies. They are presented as descriptive findings of this review, not as part of a specific model or framework.

- Standardization Agent. A facilitator role dedicated to ensuring consistent scenario execution, scoring, and assessment conditions, typically in algorithm-rehearsal exercises.
- Instructional Guide. A facilitator's role involves guiding scenario progression, encouraging discussion, clarifying decisions, and supporting learning during scenario-based exercises.
- Performance Coach. A facilitator role dedicated to monitoring performance, offering feedback, and coaching participants during or right after the exercise.
- Expert-Led Facilitation. Facilitation by senior subject-matter experts who steer discussions, interpret operational priorities, and assist with after-action learning.
- Multidisciplinary Facilitation. Facilitation by experts from various disciplines or agencies to enhance realism, coordination across agencies, and system-wide learning.
