# Supplementary material for: Design, Facilitation, and Evaluation of Tabletop Exercises for Prehospital Mass Casualty Preparedness: Scoping Review
Source: Interact J Med Res. 2026 Oct 5;15:e96228. doi: 10.2196/96228 (PMC13637994; doi:10.2196/96228)
Supplement: Supplementary file 3 [file ijmr-v15-e96228-s003.docx]

# Multimedia Appendix 3. Metadata for Unretrieved Conference Abstracts

The following 19 records were identified during the systematic search, but were conference abstracts or proceedings for which only the abstract was available, with no corresponding full-text publication identified. These records were excluded from the final synthesis because they did not meet the eligibility criteria (full-text required). Available metadata are presented below to support transparency and reproducibility.

| **#** | **Author(s)** | **Year** | **Title** | **Source** |
| --- | --- | --- | --- | --- |
| 1 | *Randy McCamey,*  *Jennifer Yeager,* | *2018* | *Amateur radio communications in a disaster preparedness simulation When all else fails . . . amateur radio* | *Journal of Emergency Management* |
| 2 | *Emma McKim Mitchell, Ryne Ackard, Michelet David McLean Estrada, Diana Hodgson, Anita Thompson-Heisterman, Alex S Aguirre Bonilla, Cynthia Foronda* | *2020* | *A partnership to develop disaster simulations for nursing students in response to climate change: description of a programme in Bluefields, Nicaragua, and Virginia, USA* | *The Lancet Global Health* |
| 3 | *Samantha Noll, Charles Little, David Silversmith* | *2023* | *Integration of Simulation-Based Exercises and Practical Skills into a Public Health Emergency Management Curriculum* | *Prehospital and Disaster Medicine* |
| 4 | *Jannah M. Obaid,*  *Ginger Bailey,*  *Heidi Wheeler,*  *Laura Meyers,*  *Sharon J. Medcalf,*  *Keith F. Hansen,*  *Kristine K. Sanger and*  *John J. Lowe* | *2017* | *Utilization of Functional Exercises to Build Regional Emergency Preparedness among Rural Health Organizations in the US* | *Prehospital and Disaster Medicine* |
| 5 | *Amir Lotfy Rashed, Anjali Cherukuri, Rie Seu, Debayan Guha, Oark Ahmed, Andrew Restivo, Cara Taubman, Maninder Singh* | *2025* | *Comparing the Effectiveness of Simulation versus Tabletop Exercises in Skill Development of Mass-Casualty Incidents for Emergency Medicine Physicians* | *Prehospital and Disaster Medicine* |
| 6 | *M. Reilly ∙ D. Markenson ∙ J. Mandel-Ricci ∙ J. Shapiro ∙ K. Pohlman ∙ G. Contreras* | *2021* | *2 Training Emergency Physicians for Mass Casualty Triage during Mass Shooting Events: A New York City- based Pilot Curriculum* | *Annals of Emergency Medicine* |
| 7 | *Lourdes Rodriguez Rivera,*  *Cynthia Rodriguez Rivera,*  *Alberto Zabala Soler,*  *Rey Pagan Rivera,*  *Luis Rodriguez and*  *Carlos Garcia-Gubern* | *2019* | *The Use of Simulation Games and Tabletop Exercises in Disaster Preparedness Training of Emergency Medicine Residents.* | *Prehospital and Disaster Medicine* |
| 8 | *Vonetta L George, Luis De La Fuente, Andres M Sanz, Carla Thomas-Brown, Patricia Clarke-Thomas , Franky Antoine, Joycelyn Walter-Thomas, Anderson Tuitt* | *2025* | *Strengthening Medical Surge Capacities through Collaborative Efforts: Emergency Medical Team – Antigua and Barbuda Deployment at SIDS4 Conference* | *Prehospital and Disaster Medicine* |
| 9 | *Nahom Tadelle Dessia, Tigist Belete Kebede, Mesgana Befekadu G/Selassie, David John Vassallo, Jonathan Haveloch Barden, Mai Ahmed Attia Seida, Rosemary Nkiru Emodi, Neima Ali Zeinu* | *2025* | *HOSPEX Tabletop Simulation for Emergency Medical Teams: Ethiopia Case Study* | *Prehospital and Disaster Medicine* |
| 10 | *Anna O'Leary, Deirdre Breslin, Jeffrey Mulcaire, Meabh Eager, Jean O'Sullivan* | *2023* | *Participants' Experience of Completing Trauma and Critical Skills Training in a Resource-Limited Environment* | *Prehospital and Disaster Medicine* |
| 11 | *Deirdre Breslin, Jeffrey Mulcaire, Anna O'Leary, Roy McKenna, Bronagh MacManus, Jean O'Sullivan* | *2023* | *Exploring Irish Faculty Members' Experiences of Delivering a Multi-modal Medical Education Course in a Resource-limited Environment* | *Prehospital and Disaster Medicine* |
| 12 | *Geoffrey L. Shapiro, John H. Armstrong, Kathryn Roberts, James Gordon, E. Reed Smith, Jeffrey Afman, Scott M. Sasser* | *2019* | *The Joint Counterterrorism Awareness Workshop Series (JCTAWS): Integrating disciplines for enhanced capabilities during a complex coordinated attack* | *Journal of Emergency Management* |
| 13 | *Samuel E. Shartar, MSN, RN, Brooks L. Moore, MD, Lori M. Wood MSEM* | *2017* | *Developing a Mass Casualty Surge Capacity Protocol for Emergency Medical Services to Use for Patient Distribution* | *Southern Medical Journal* |
| 14 | *Heejun Shin, Attila Hertelendy, Alexander Hart, Fadi Issa, Gregory Ciottone* | *2023* | *Evaluating the Educational Effectiveness of Hospital Healthcare Providers after Chemical Mass Casualty Response Training Program.* | *Prehospital and Disaster Medicine* |
| 15 | *Eric Weinstein, Michelangelo Bortolin, Hamdi Lamine, Teri Lynn Herbert, Ives Hubloue, Sofie Pauwels, Rita Burke, Mark Cicero, Phoebe Dugas, Elizabeth Oduwole* | *2023* | *The Challenge of Mass Casualty Incident Response Simulation Exercise Design and Creation: A Modified Delphi Study* | *Prehospital and Disaster Medicine* |
| 16 | *Skaltsis, Jessica; Sackett, Ashlee; Ellis, Kerri A.; Cohen, Steven A.* | *2024* | *Learner Perception of Disaster Simulation Modalities: A Pilot Study* | *Nurse Educator* |
| 17 | *Tang, L.-N.; Li, M.; Huang, H.-D.; Quan, Y.; Li, B.; Jia, W.-R.; Yang, Y.* | *2021* | *Combat casualty care training of Chinese peacekeeping military doctors: An evaluation of effectiveness* | *Academic Journal of Second Military Medical University* |
| 18 | *Voinschi, R.; Chesov, I.; Stetsyk, V.* | *2024* | *A Framework to Strength National Anesthesia and ICU Services Preparedness to Response to Mass Casualty Incidents in the Republic of Moldova* | *Anesthesia and Analgesia* |
| 19 | [Unknown authors] | *2025* | *The Effect of Multidimensional Disaster Education on Disaster Awareness and Disaster Response Self- Efficacy Among Student Nurses Affected by the Kahramanmaraş-centered Earthquake: a Quasi- Experimental Randomized Controlled Trial* | *MedPath* |
