# Supplementary material for: Design, Facilitation, and Evaluation of Tabletop Exercises for Prehospital Mass Casualty Preparedness: Scoping Review
Source: Interact J Med Res. 2026 Oct 5;15:e96228. doi: 10.2196/96228 (PMC13637994; doi:10.2196/96228)
Supplement: Supplementary file 4 [file ijmr-v15-e96228-s004.docx]

# Multimedia Appendix 4. Kirkpatrick Outcome Mapping by Study and Exercise Tier

Outcomes classified using Kirkpatrick’s Evaluation Model with Level 2+ (Applied Learning) subcategory. Studies organized by TTX Design Spectrum tier.

Level 2+ (Applied Learning): Structured performance assessment within the exercise environment, including triage accuracy scoring, observed team behaviors, decision quality, and incident command compliance measured via standardized instruments.

Level 3 (Behavior): Documented application of exercise-derived skills in authentic operational contexts (real-world MCI responses or verified practice changes).

— indicates level not assessed in that study.

| Study | Level 1 Reaction | Level 2 Learning | Level 2+ Applied Learning | Level 3 Behavior | Level 4 Results |
| --- | --- | --- | --- | --- | --- |
| Tier 1: Algorithm Rehearsal | | | | | |
| Cheng et al (2022) [25] | Perceived usefulness, usability | — | Triage accuracy scoring across six pediatric triage algorithms (CareFlight, SALT, JumpSTART, PTT, SMART, Sacco) against reference standards | — | — |
| McGlynn et al (2020) [26] | — | — | Triage accuracy scoring (SALT algorithm) against reference standards for 10, 100, and 1000 pediatric patient sets | — | — |
| Tier 2: Scenario-Based Decision Training | | | | | |
| Kim et al (2021) [27] | Usability, satisfaction | Knowledge test, confidence | Structured observation of performance across triage, decontamination, zoning, resource allocation, and ICS operations during exercise scenarios | — | — |
| Phattharapornjaroen et al (2020) [28] | — | Self-reported knowledge and understanding | Observed leadership behaviors and teamwork during tabletop exercises; creation of communication and coordination processes; scenario management across exercises | — | — |
| Sena et al (2021) [4] | Perceived usefulness, satisfaction | Perceived preparedness (self-reported competency), knowledge test, confidence | — | — | — |
| Farhat et al (2022) [29] | Perceived usefulness, satisfaction, relevance | Knowledge test | Scenario-based tasks performed during tabletop exercise (CBRNE response competencies) | — | — |
| Alakrawi et al (2024) [30] | Perceived usefulness | Perceived knowledge and understanding, confidence | — | — | — |
| Chumvanichaya et al (2025) [31] | Attention, confidence, satisfaction, relevance | Knowledge test (including 1-week delayed post-test) | Triage performance accuracy and decision-making speed during exercise scenarios | — | — |
| Cuartas-Alvarez et al (2026) [32] | Perceived usefulness | Self-reported knowledge and understanding, knowledge test | — | — | — |
| Tier 3: Systems Integration Testing | | | | | |
| Anan et al (2016) [33] | — | — | After-action report compliance during sequential CBRNE scenarios | — | — |
| Cicero et al (2019) [34] | Perceived usefulness, satisfaction, relevance | Knowledge test | — | — | Adopted pediatric preparedness checklist; formalized new MOUs; conducted additional pediatric MCI drills at 2-year follow-up |
| Skryabina et al (2020) [35] | Satisfaction, perceived usefulness | Confidence, knowledge test, attitude | — | Documented application of exercise-derived knowledge and decision-making patterns during the Manchester Arena bombing response; improved teamwork; correct use of casualty distribution plan; real-time adjustments based on prior learning | System-wide improvements in casualty distribution and operational efficiency during the actual MCI response, attributed by participants and planners to prior exercise learning (uncontrolled observation); reduction of previously identified response barriers |
| Zimmerman et al (2026) [36] | Satisfaction, perceived usefulness | Self-reported knowledge and understanding, knowledge test | Participants observed/scored during exercises using CSCATTT-anchored instructor observation | Participants later assumed preparedness coordinator and instructor-candidate roles and continued preparedness work beyond the exercise environment | A structured simulation platform, continued iterations/refresher activity, and departmental preparedness/capacity-building effects |
| Total (N=13) | n=10 | n=10 | n=8 | n=2 | n=3 |

ICS = Incident Command System; MCI = Mass Casualty Incident; MOU = Memorandum of Understanding; PTT = Pediatric Triage Tape.
