# Supplementary material for: Design, Facilitation, and Evaluation of Tabletop Exercises for Prehospital Mass Casualty Preparedness: Scoping Review
Source: Interact J Med Res. 2026 Oct 5;15:e96228. doi: 10.2196/96228 (PMC13637994; doi:10.2196/96228)
Supplement: Supplementary file 5 [file ijmr-v15-e96228-s005.docx]

# Multimedia Appendix 5. Practitioner Summary: Purpose-Aligned Design, Facilitation, and Evaluation by TTX Tier

This summary presents a practitioner-facing table that consolidates purpose–facilitation–evaluation pairings from the 13 selected studies. It includes no new information; all data are from the Results, Tables 2 and 3, and Multimedia Appendices 3 and 5. Aimed at exercise commissioners, EMS training officers, and preparedness planners, it complements HSEEP-style exercise objectives and Exercise Evaluation Guides instead of replacing them.

|  | Tier 1: Algorithm Rehearsal | Tier 2: Scenario-Based Decision Training | Tier 3: Systems Integration Testing |
| --- | --- | --- | --- |
| Interaction level rehearsed | Individual responder ↔ response protocol | Interdisciplinary team members ↔ one another, under scenario constraints | Organizations, agencies, and systems ↔ one another |
| Typical objectives | Proficiency and accuracy in a specific protocol (usually triage); standardized measurement of individual performance | Integrated multi-competency decision-making: incident command, team coordination, resource allocation, communication, CBRNE response | Interagency coordination, organizational readiness, role clarification, policy and plan refinement |
| Matched facilitation mode(s) | Standardization Agent (rater calibration, scripted delivery, standardized scoring) | Instructional Guide (timed injects, prompts, structured debriefing); Performance Coach (embedded observers giving iterative feedback) | Expert-Led (senior clinicians/preparedness experts leading after-action review); Multidisciplinary (multi-agency facilitation teams) |
| Proportionate evaluation target | Level 2+ in-exercise performance: decision accuracy against reference standards, speed, inter-rater reliability. Level 1 optional. Claims of organizational impact not expected. | Level 1 (reaction) + Level 2 (knowledge, task-specific self-efficacy) + Level 2+ (structured observation of team performance). If practice change is intended, add a follow-up method: delayed testing, repeat scenarios, structured interviews, or practice audits. | Level 2+ during the exercise (e.g., after-action report compliance) plus Level 3/4 follow-up: documented role application, policy or MOU changes, preparedness-metric improvement. |
| Example instruments from the included studies | Triage accuracy and speed scoring against reference standards across 10–1000-victim sets (McGlynn et al, 2020 [26]) and six algorithms (Cheng et al, 2022 [25]) | Pre/post knowledge tests; structured observation of triage, decontamination, zoning, resource allocation, and ICS operations (Kim et al, 2021 [27]); embedded-supervisor observation of leadership and teamwork (Phattharapornjaroen et al, 2020 [28]); one-week delayed post-test (Chumvanichaya et al, 2025 [31]) | After-action report compliance during sequential scenarios (Anan et al, 2016 [33]); hospital preparedness checklist with 2-year follow-up (Cicero et al, 2019 [34]); Kirkpatrick paired with the PARIHS implementation framework (Skryabina et al, 2020 [35]); CSCATTT-anchored instructor observation (Zimmerman et al, 2026 [36]) |
| Suggested data elements to record | Protocol and version; per-case triage decision vs reference standard; accuracy, over-/under-triage rates; decision time per case; rater identity and calibration/reliability statistics | Scenario and inject log; targeted competencies; observation-rubric scores by competency; knowledge/self-efficacy pre/post scores; debriefing themes; follow-up assessment results and interval | Participating agencies and roles; coordination gaps identified; after-action findings, corrective actions, and owners; policy, plan, or MOU changes; preparedness metrics at defined follow-up intervals |

Abbreviations: CBRNE = chemical, biological, radiological, nuclear, explosive; CSCATTT = Command, Safety, Communication, Assessment, Triage, Treatment, Transportation; HSEEP = Homeland Security Exercise and Evaluation Program; ICS = Incident Command System; MOU = memorandum of understanding; PARIHS = Promoting Action on Research Implementation in Health Services; TTX = tabletop exercise.
