# Supplementary material for: Design, Facilitation, and Evaluation of Tabletop Exercises for Prehospital Mass Casualty Preparedness: Scoping Review
Source: Interact J Med Res. 2026 Oct 5;15:e96228. doi: 10.2196/96228 (PMC13637994; doi:10.2196/96228)
Supplement: Supplementary file 6 [file ijmr-v15-e96228-s006.docx]

# PRISMA-ScR checklist

Preferred Reporting Items for Systematic Reviews and Meta-Analyses Extension for Scoping Reviews (PRISMA-ScR)

Tricco AC, Lillie E, Zarin W, et al. Ann Intern Med. 2018;169(7):467-473.

| Item | PRISMA-ScR Checklist Item | Reported in Section |
| --- | --- | --- |
| Title | | |
| 1 | Identify the report as a scoping review | Title (“A Scoping Review”) |
| Abstract | | |
| 2 | Provide a structured summary including background, objectives, eligibility criteria, sources of evidence, charting methods, results, and conclusions | Abstract (Background, Objective, Methods, Results, Conclusions) |
| Introduction | | |
| 3 | Describe the rationale for the review in the context of what is already known; explain why the questions/objectives lend themselves to a scoping review approach | Introduction, paragraphs 1–5 (dispersed evidence; comparison with prior broader reviews) |
| 4 | Provide an explicit statement of the questions and objectives with reference to key elements (population, concepts, context) | Introduction, final paragraph; Methods, Stage 1 |
| Methods | | |
| 5 | Indicate whether a review protocol exists; state if and where it can be accessed; provide registration information | Methods, Study design (Protocols.io, registered July 3, 2025 [14]; preprint [15]) |
| 6 | Specify characteristics of the sources of evidence used as eligibility criteria (years, language, publication status) and provide a rationale | Methods, Eligibility criteria (January 2015–May 2026; English; peer-reviewed or grey literature; rationale stated) |
| 7 | Describe all information sources in the search and the date the most recent search was executed | Methods, Stage 2 (seven databases/registers, Google Scholar, reference lists; strategy developed June 10, 2025; updated May 15, 2026) |
| 8 | Present the full search strategy for at least one database such that it could be repeated | Table 1 (representative terms); Multimedia Appendix 2 (complete database-specific strings, including full PubMed strategy) |
| 9 | State the process for selecting sources of evidence (screening and eligibility) | Methods, Stage 3 (dual independent two-step screening in Covidence; third-reviewer adjudication) |
| 10 | Describe the methods of charting data and any processes for obtaining and confirming data from investigators | Methods, Stages 2 and 4 (standardized form piloted on five studies; dual independent extraction; no investigator contact) |
| 11 | List and define all variables for which data were sought and any assumptions and simplifications made | Methods, Stage 4 (study details, TTX features, facilitation, evaluation methods, Kirkpatrick levels, frameworks); Multimedia Appendix 3 (classification rules) |
| 12 | Describe the methods of handling and summarizing the data | Methods, Stage 5 (descriptive and narrative synthesis across seven domains; TTX Design Spectrum typology) |
| 13 | State any critical appraisal of individual sources of evidence (if applicable) | Not conducted, consistent with scoping review methodology; stated in Limitations |
| 14 | Describe methods of handling and summarizing data from critical appraisal (if applicable) | Not applicable (no critical appraisal conducted) |
| Results | | |
| 15 | Give numbers of sources of evidence screened, assessed for eligibility, and included, with reasons for exclusions, ideally using a flow diagram | Results, Literature search (2713 identified [2693 databases/registers + 20 other sources] → 2441 screened → 88 sought → 69 assessed → 13 included; 56 excluded: wrong setting n=22, wrong intervention n=21, wrong study design n=11, non-English n=2); Figure 1 |
| 16 | For each source of evidence, present characteristics for which data were charted and provide the citations | Results, Study characteristics; Table 2; Multimedia Appendix 5 |
| 17 | Present the main results as they relate to the review questions and objectives | Results (TTX Design Spectrum; facilitation approaches; evaluation strategies and Kirkpatrick mapping, Table 3; frameworks); Figures 2–5 |
| Discussion | | |
| 18 | Summarize the main findings, including the evidence for each principal finding; discuss relevance to key groups | Discussion (Principal findings; Separating applied learning from behavioral transfer; Timing, transfer, and facilitation; Frameworks and implementation); Figure 6 |
| 19 | Discuss the limitations of the scoping review process | Limitations |
| 20 | Provide a general interpretation of the results with respect to the review questions and objectives, and potential implications and/or next steps | Discussion (practice implications); Future research; Conclusions |
| Funding | | |
| 21 | Describe sources of funding for the included sources of evidence and for the scoping review, and the role of the funders | Funding statement (MBRU Dubai Health Collaborative Stimulus Research Grant, CSRG, for the MCIPHER project; funder had no role). Funding of the included sources of evidence was not extracted. |
| 22 | Indicate whether a protocol for the review was registered and provide registration details | Methods, Study design (Protocols.io, July 3, 2025 [14]; preprint [15]) |
